# Co-production of a nature-based intervention for children with ADHD study (CONIFAS): Protocol for co-production phases

**DOI:** 10.1371/journal.pone.0274375

**Published:** 2022-09-20

**Authors:** Hannah A. Armitt, Ellen N. Kingsley, Leah Attwell, Piran C. L. White, Kat Woolley, Megan Garside, Natasha Green, Michael Hussey, Peter A. Coventry

**Affiliations:** 1 Research and Development Department, Humber Teaching NHS Foundation Trust, Willerby, United Kingdom; 2 Child Oriented Mental Health Innovation Collaborative, Leeds and York Partnership NHS Foundation Trust, York, United Kingdom; 3 Department of Environment and Geography, University of York, York, United Kingdom; 4 York Environmental Sustainability Institute, University of York, York, United Kingdom; 5 Yorkshire Wildlife Trust, Yorkshire, United Kingdom; 6 Patient and Public Involvement Lead, York, United Kingdom; 7 ADHD Foundation, Liverpool, United Kingdom; 8 Department of Health Sciences, University of York, York, United Kingdom; 9 Leverhulme Centre Anthropocene Biodiversity, University of York, York, United Kingdom; Public Library of Science, UNITED KINGDOM

## Abstract

Children with Attention Deficit Hyperactivity Disorder can face difficulties with inattention, hyperactivity, and impulsivity, which can impact many areas of their lives, including their educational attainment and social and emotional wellbeing. Involvement in nature-based activities can reduce these difficulties and improve wellbeing, but there are limited resources for supporting children with this diagnosis to access these approaches and no nature-based interventions designed with and for this group. This protocol describes a co-production study in which children diagnosed with Attention Deficit Hyperactivity Disorder aged 5–11 years old, their parents/guardians, and professionals will attend a series of workshops to share their knowledge to co-produce a new nature-based intervention for this population of children. We aim to understand how the children’s’ experiences of Attention Deficit Hyperactivity Disorder may affect their interactions with nature, identify how activity in and with nature may help with symptom reduction and general wellbeing, and co-produce an intervention for families which applies our learning. The result of this study will be the designed intervention and insights into how children and young people with Attention Deficit Hyperactivity Disorder interact with nature. The prototype intervention will then undergo feasibility testing in a future study.

**Trail registration:** NIHR203043; ISRCTN11763460.

## Introduction

### Background

Attention Deficit Hyperactivity Disorder (ADHD) is a neurodevelopmental condition which is estimated to affect approximately 5% of children and adolescents globally [1]. In the UK 2% of adults and between 3–5% of children in the UK have ADHD [2]. It is characterised by symptoms of inattention, hyperactivity, and impulsivity, each of which can have a significant impact on daily functioning. Children with ADHD can experience difficulties with concentration and are frequently over-active regardless of setting. Additionally, they often have comorbid mental health difficulties (e.g. autism, speech and language difficulties, anxiety, low mood), poorer social and emotional wellbeing and educational outcomes, and an increased likelihood of relationship breakdowns [3].

Early identification and support can prevent the development of further mental health conditions for those with ADHD and can have a positive impact on quality of life. The current study is set within the context of the UK and the healthcare provision provided by the National Health Service (NHS). This provision, particularly within the context of mental health care, does not sufficiently meet the needs of this population [4]. Long waiting lists for access to treatment through Child and Adolescent Mental Health Services (CAMHS) mean that currently 6% of families wait over 12 weeks for a first appointment with CAMHS and a further 48% of families have their referrals closed before treatment is offered [5]. It is also reported that at time of diagnosis, families do not routinely receive appropriate intervention [4] which can lead to deterioration in the child’s wellbeing.

ADHD can also have a negative impact on the parents or guardians and informal caregivers of children with ADHD. The Caregiver Perspective on Paediatric ADHD study reported 38% of caregivers (n = 2872) had been late for work in the past month because of their child’s ADHD, and that 31% of caregivers (n = 3688) had altered their employment status [6]. Telford et al [7] identify the need to develop and evaluate early interventions which have the potential to reduce the longer-term burden of ADHD.

Green spaces such as parks, allotments, and woodlands are increasingly recognised as ‘natural capital’ that offer public health benefit for both adults and children [8]. These health benefits can extend to children with ADHD. According to a study reported in The Lancet, increased exposure to green spaces for children is strongly associated with lower rates of ADHD diagnoses and occurrence of ADHD symptoms [9]. Furthermore, improvements are seen in attention restoration, memory, stress and academic test scores [10], as well as reduced need for medication [11]. Green and blue spaces may help with ADHD symptoms in several ways, for example through availability of space and the associated benefits of physical activity to expend excess energy [12]. Natural spaces can provide a multi-sensory space, removing external distractions such as technology, and increasing the ability to stay focused [13]. The relaxing effects of natural spaces can also impact on behavioural patterns often associated with ADHD, including impulsivity, inattention, aggression, and reduced sociability [14] Moreover, children with ADHD have reported that engaging in outdoor activity which incorporates nature and is done with others makes their life “really good”, i.e. is important for their life satisfaction [15].

Children who experience social and economic deprivation are more likely to be diagnosed with ADHD [16] and have less access to green space [17, 18]. Furthermore, much of the evidence about green space and health benefits for children with ADHD is premised on exposure rather than active participation in nature. Evidence consistently shows the benefits for children and young people of engaging with nature for their physical activity levels, health outcomes, increased wellbeing, reduced stress, and development of a positive affinity with nature [19]. While nature-based interventions align with National Institute for Health and Care Excellence (NICE) guidelines [2] for children with ADHD there are no well evidenced nature-based interventions for use in the community or in NHS Child and Adolescent Mental Health services (CAMHS).

### Rationale

The CO-production of a Nature-based Intervention For children with ADHD Study (CONIFAS) aims to co-produce a nature-based intervention for use with families of children and young people with ADHD to reduce the impact of ADHD symptoms and improve quality of life. Co-production has been chosen as the methodology as its central value is the development of more equal partnerships between people who use services and people who design and implement them. Co-production has been linked with better outcomes for people who use services and higher levels of accessibility, acceptability, and engagement [20–22]. Co-produced mental health interventions can also be created fairly quickly and for a relatively low cost [23].

The prototype intervention will be developed in the first instance to be used in the NHS by Child and Adolescent Mental Health services (CAMHS). Throughout co-production, the study team will work closely with child and adolescent mental health professionals and other stakeholders in ADHD services and nature-based recreation (e.g. education and charity professionals). This will ensure the creation of links and knowledge-sharing between the end users (families of children and young people with ADHD), local mental health services, and those with expertise in delivering nature-based activities. Additionally, links made between a local wildlife charity partner (Yorkshire Wildlife Trust) and the ADHD Foundation will add value to current practice and knowledge.

## Materials and methods

Ethical approval for this study was granted by the University of York’s Department of Environment and Geography Research Ethics Committee (REC) on 23/05/2022 (S1 File). This study will be conducted in accordance with ICH Good Clinical Practice guidelines. The study is registered with ISRCTN (11763460) and any changes to this protocol will be reported in the ISRCTN registry [24].

### Objectives

**Fig 1:**
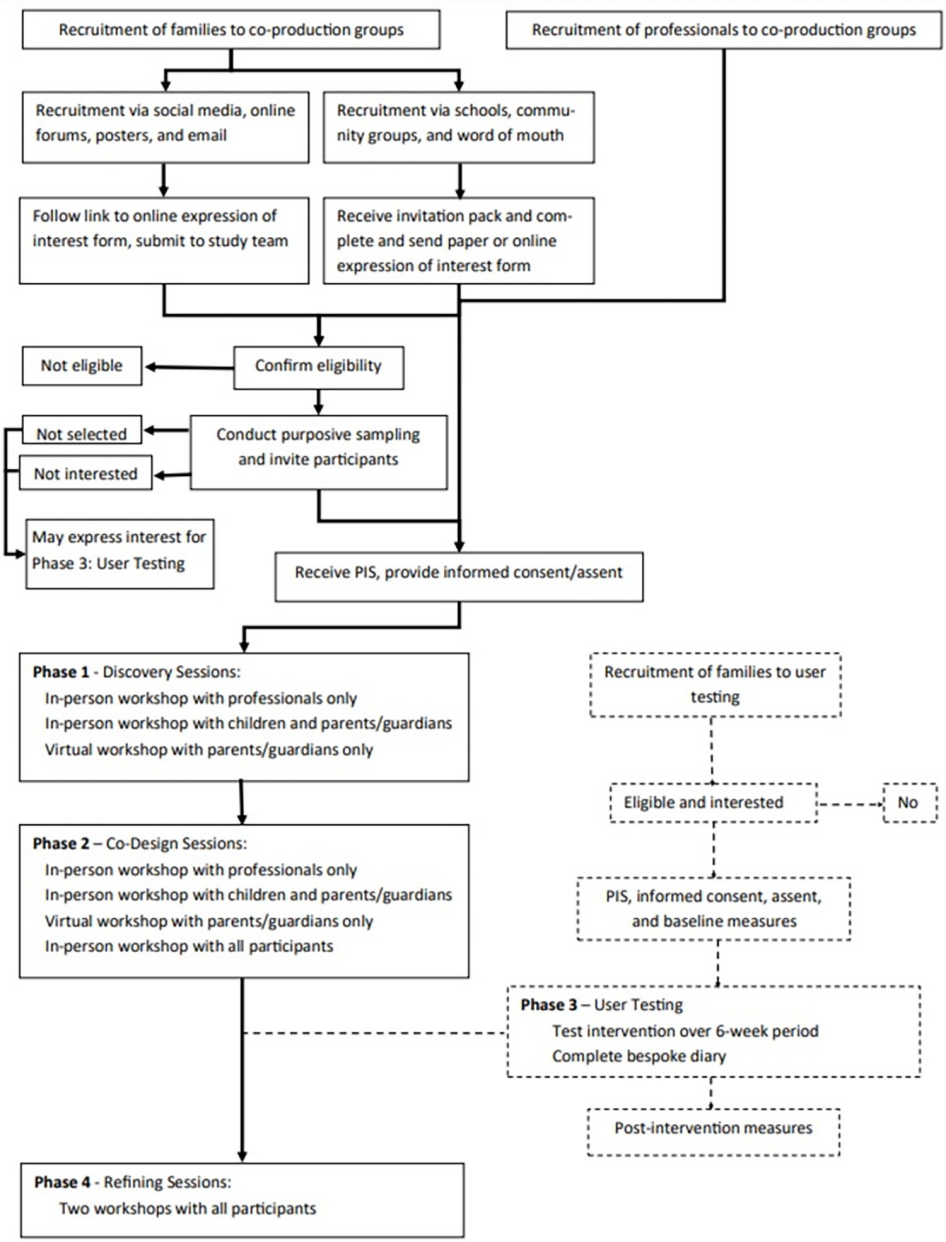
Flow diagram of study components and phases. PIS: Participant information sheet.

The CONIFAS study consists of two elements: co-production of a nature-based intervention, and user testing of the co-produced intervention. These elements will be based on the Design Council’s Double Diamond model (Design Council, 2017): Discover, Define, Develop, and Deliver. These phases will map on to four phases of the research study, each of which has a specific objective: Phase 1 will involve discovery workshops in which we aim to understand the problem (objective 1); phase 2 will involve co-production workshops to define the intervention (objective 2); phase 3 will involve user testing to test and develop the intervention (objective 3); and phase 4 will include refinement of the intervention with the co-production groups based on outcomes from phase 3, ready for feasibility testing (objective 4). The flow and sequence of the study components are shown in Fig 1.

Three of the above listed phases are part of the co-production element of the study and one is part of the user testing element. This protocol reports the methods for three of the four phases, the ones which make up the co-production element of the trial (phases 1, 2, and 4 which map onto objectives 1, 2, and 4). The co-production element is not considered to be research as no data will be collected. Phase 3 and objective 3, which make up the user testing element, will be covered by a separate protocol and ethical review once the prototype intervention has been created. The user testing phase is considered to be research as data will be collected.

The study objectives are:

1. Create a co-production team of parent/guardian-child dyads with lived experience of ADHD, voluntary organisations working in green spaces, NHS professionals, clinicians, education professionals, and researchers. Investigate the strengths and difficulties associated with an ADHD diagnosis in children and how nature can be used to support affected children (Phase 1).
2. Develop an appropriate and acceptable intervention for families of children and young people with ADHD through discovery and co-production workshops, using existing campaigns (such as the five ‘Ways to Wellbeing’ and Wildlife Trust’s 30 Days Wild) and resources for inspiration (Phase 2).
3. Recruit a further 10 families to conduct a user testing trial to test the usability, acceptability, and accessibility of the created intervention (Phase 3). This data collection element of the study is detailed in a separate protocol.
4. Refine the intervention with co-production participants from phases 1 and 2 using feedback from the user testing (Phase 4).

### Patient and public involvement

**Fig 2:**
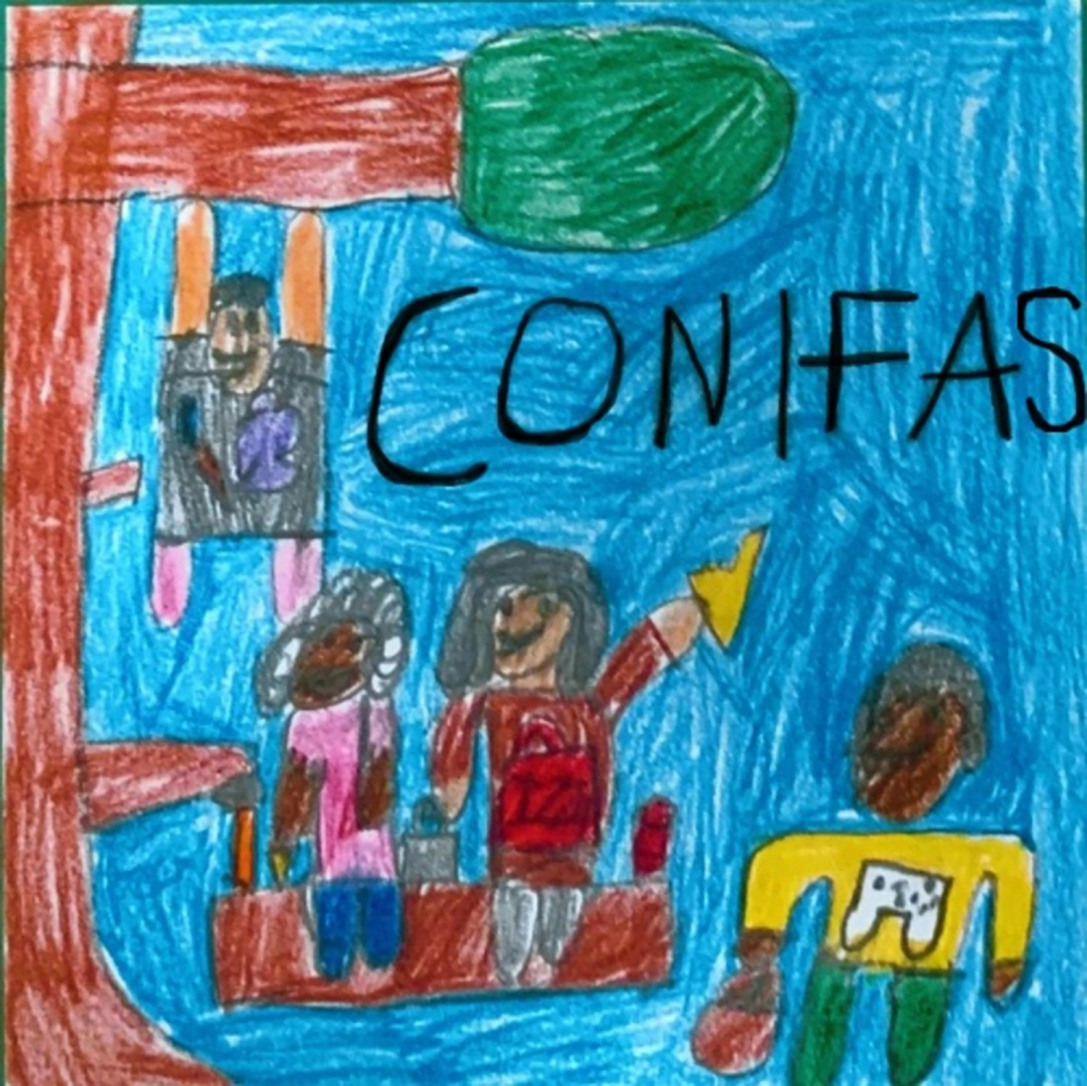
CONIFAS study logo based on the winning entry to the drawing competition.

Patient and public involvement (PPI) is central to CONIFAS. A parent of a child with ADHD, a professional from the ADHD foundation, and two professionals from the Yorkshire Wildlife Trust are co-investigators on the study. They have been involved in the development stages and they will be involved for the duration of the study. Key responsibilities for this PPI group will be to review all participant-facing documents and contribute to the Study Management Group (SMG). Participant-facing documents have also been reviewed by the sponsor’s (LYPFT NHS) research patient ambassador. We also enhanced inclusivity and reach of our PPI strategy by holding a picture drawing competition for children and young people and this image is used to promote the visual identity of the study (Fig 2).

### Participants

Participants will be children with ADHD, their parent or guardian, and professional stakeholders in ADHD (e.g., professionals from CAMHS, education, young people’s charities) who live in the local areas (Yorkshire and the Humber, which encompasses York, East Riding of Yorkshire, South Yorkshire, West Yorkshire, and North Yorkshire counties). For the co-production phases (1, 2, 4), a total of 30 participants will be recruited: 10 children, their parent/guardian, and 10 professionals.

#### Eligibility criteria

Participating children and young people must: be aged between 5–11 years; have a diagnosis of ADHD as reported by their parents/guardians; and have sufficient understanding of English to participate in the co-production events and/or the intervention testing. Children who pose a risk of harm to themselves or others and children who would not be able to participate in the co-production groups due to profound additional difficulties as determined by parents/guardians will not be eligible to take part.

Participating parents/guardians and professionals must also have sufficient understanding of English to participate in the co-production events and/or the intervention testing.

All participants will live or work in the area local to York and be able to travel to the designated meeting location: Barlow Common, Selby, North Yorkshire.

### Recruitment

Families will be identified via social media (including our websites [comic.org.uk; www.york.ac.uk/healthsciences/research], Twitter, and relevant parenting forums), posters, local council advertisements, schools, and community groups. We will work with our networks (NHS staff, the University of York and York Environmental Sustainability Institute, green social prescribing champions, research inclusivity champions e.g. the Ethnic Minority Research Inclusion group, and local national park contacts), researcher groups, and known community groups to try to recruit underrepresented families by promoting our study to wider audiences. A mixture of professional stakeholders in ADHD from different fields will be recruited through northern and regional CAMHS, third sector, and education settings via established contacts in these organisations, email, and telephone contact utilising existing links. These links include professional networks of co-investigators and ongoing and previous research studies conducted by the team.

Interested parents/guardians will complete an expression of interest survey which includes a short demographic questionnaire containing questions about identity, family structure, and locality. The intention of this procedure is to capture the needs of children across the specified age range, diverse cultures, large and small families, parent/guardian education levels, and location types (urban, rural, and coastal locations) to co-produce an intervention which meets the needs of a diverse range of life experiences. Recruitment will be opportunistic, but we are working with partners and stakeholders who can offer access to a diverse range of potential participants.

If we do not receive a large number of EOIs by an agreed-upon date and are unlikely to meet the recruitment target, the study team will cease the use of this tool for recruitment purposes. Eligible families will be recruited by approaching the study team directly and will be asked to complete the EOI for the purposes of consent to retain contact details rather than for collection of demographic details.

All interested participants will receive appropriate and ethically approved participant information sheets and will be given sufficient time to review these and ask any questions. If participants wish to proceed, professionals will be asked to provide informed consent, and parents/guardians will be asked to provide informed consent both for themselves and on behalf of their child. Children will complete an assent form where appropriate.

### Sample size and data

It is intended that a total of 30 participants will be recruited for the co-production part of this research study: 10 children, one parent/guardian for each child, and 10 professionals. Group size is based on guidance for co-production methodology that allows for varied views but equal participation [25].

### Study activities—workshops

The three phases of this research study which constitute the co-production element (phases 1, 2, and 4) will be carried out via a series of workshops described below. Recruited participants will be asked to attend all workshops to ensure continuity of input. All workshops will be attended by members of the research team, the Yorkshire Wildlife Trust, and a graphic designer who will create visual notes from each workshop which will then be shared with subsequent workshops. An alternate activity will be offered in cases where families need to bring participating children’s siblings to the workshops. Families will be given a £20 Love2Shop voucher after each in person workshop as well as a ‘goodie bag’ (e.g. stickers, pencil, small magnifying glass for insect observation) for children to take home. Lunch will be provided for all participants at each in-person workshop and travel expenses will be reimbursed. Waterproof and cold-weather clothing will be available for any participants who need them.

#### Pre-workshop

Workshops will be designed to be positive and accessible experiences for participants. At the point of recruitment, we will offer the ADHD Hero Activity Book [26] to families as a supplementary source of positively framed information on the impacts of ADHD. This booklet contains a series of activities which help children to understand their diagnosis and how they can be supported and seek support. This resource was also co-produced with children and families. Although we will not require that the workbook be used or completed, this resource will provide a piece of psychoeducation before families attend the workshops and serve to dispel any myths about ADHD, whilst also setting a positive tone ahead of the workshops. We are aware that discussing a disorder such as ADHD with young children and families may be difficult, that children may not be able to articulate their feelings, and that children may not be entirely aware of their diagnoses and its impacts. We therefore do not want to focus on any deficits or to make children feel negatively about themselves, but instead to celebrate the positives and talk about the challenges in a solution-focused way.

We will also use the Iriss Inclusion Checklist [27] for families before the sessions to maximise accessibility. ADHD often coincides with various co-morbidities such as autism, dyslexia, anxiety, and motor difficulties and we want to ensure that children and parents/guardians with non-excluding difficulties are supported to participate.

#### Phase 1 –Discover

The discovery phase aims to understand and define ADHD and its symptomatology for children, the potential methods of improving symptoms, any barriers, the role of nature-based activities in this, and which nature-based activities may be helpful for children with ADHD. This will occur over three separate workshops:

1. An in-person, half-day event with professional stakeholders in ADHD services.
2. An in-person, half-day event with children and their parent/guardian to gather children’s views and for them to participate in some preliminary nature-based activities.
3. An online workshop with just parents/guardians to gather parent/guardian views.

Our session activities and co-production methodology have been further informed by the Diversity for Design Framework for ADHD developed by Fekete and Lucero [28]. All workshops will be structured to facilitate co-production. For example, participants will be encouraged to bring their own resources to lead sharing and discussion, ‘teacher-classroom’ structuring will be avoided by sitting/standing together, and equal roles will be encouraged for everyone involved. Professional sessions will use different prompting questions but will follow a similar structure to the parent/child sessions with experiential nature-based breaks included. To support the children, we will use a visual schedule at every session which will incorporate break times.

At the beginning of each workshop, we will set our priorities together. This will include describing the ground rules, ensuring that everyone feels included, helping everyone to understand their role, and understanding what we aim to achieve at each session and how. The child and parent/guardian sessions will start with initial icebreaker activity, for example designing a name badge. We will then move on to two rounds of nature-based activities to allow participants to experience and discuss the types of activities which might make up the intervention.

#### Phase 2 –Define

This phase will focus on the development of the intervention ready for user testing. It will likely involve refinement of the activities suggested and discussed in phase 1. We will use existing resources to facilitate our discussions, such as the Yorkshire Wildlife Trust 30 Days Wild programme, and the New Economics Foundation’s ‘5 Ways to Wellbeing’. This will occur across four workshops:

1. An in-person, half-day event with professionals.
2. An in-person, half-day event with children and parents/guardians.
3. An online workshop with parents/guardians.
4. An in-person, half-day event with all participants.

#### Phase 3 –Develop

Phase 3 will involve recruiting 10 new child and parent/guardian dyads to conduct user testing of the designed intervention. This phase will be described in further detail in a future protocol developed following phases 1 and 2 of the co-production part of this research study.

#### Phase 4 –Deliver

In the final phase, all 30 participants from phases 1 and 2 will attend two workshops to look at the results of the user testing phase, refine and make any changes to the intervention, and finalise the intervention and any intervention materials for wider use.

#### Missed activities and participant contact

If a participant misses a workshop but wishes to continue in the study, they will be offered a phone/video call with a member of the research team to go through the summary of the day and to add any of their thoughts, experiences, or ideas.

We will also agree with each participant group (parent/child dyads and professionals) how they may like to keep in contact with the research team in-between sessions, should they like to. This may include emailing, the use of online knowledge-sharing tools (e.g. Google Jamboard or WhatsApp groups) with the purpose of sharing any relevant information, experiences, or ideas in the meantime. These reflections will be discussed at the next available workshops. Participant contact details will not be shared by the research team.

#### Safety and risk

We do not anticipate that participants will be subject to any substantial risks during this study. However, the focus of this study is on nature-based and outdoor activities, with workshop sessions involving participation in these activities and being outdoors (weather dependant). As such, usual risks associated with being outdoors may be expected including but not limited to slips, trips, sunburn, insect bites and stings. All children will be attending with a parent/guardian or carer who will remain responsible for their child. All participants will be reminded of the risks, encouraged to proceed with caution, notified of any particular risk areas (e.g. slippery ground) and reminded to attend sessions wearing appropriate footwear and clothing with plenty of notice. Where needed, additional clothing, particularly for cold or wet weather, will be provided by Yorkshire Wildlife Trust on site. A risk assessment of the Yorkshire Wildlife Trust site and the planned activities will be conducted, and experienced Yorkshire Wildlife Trust staff members will be present at each event.

Participants will be asked to complete a risk management form where they can report known risks. Participants will be reminded before each session to bring any relevant medication including inhalers and emergency medication. It will be the responsibility of the participants (or their attending parents/guardians) to manage and administer their own medications.

Lunch will be provided for participants when they attend an in-person session. All participants and attending staff members will be asked to report any allergies and intolerances to the study team, and these will be catered for accordingly.

The research team recognise that children with ADHD may find it hard listening to and retaining instructions due to the nature of the disorder. The researchers will be advised by experienced co-investigators who work with this population and will ensure that instructions, particularly pertaining to any risks, are clearly communicated in an accessible way and that parents/guardians, carers, and staff are attentive to the children.

### ‘Data’ and data capturing

We will not record workshops nor qualitatively analyse any data using formal methodology, as we feel this will not be indicative of true co-production. Notes will be taken on the day by researcher observers and visual notes will be created by a graphic designer. At the end of each session the co-production group will go through the notes together and collaboratively agree on what our main conclusions/takeaways for each session are. Notes from previous sessions will then be available at subsequent sessions somewhere that they can be seen by all participants. This will include the visual notes created by the graphic designer and researcher notes in different formats, all of which will be easy to read and child friendly.

## Results and discussion

This study is currently open for recruitment of families and professionals for the co-production phases of the research. Recruitment for this phase will conclude on the 19^th^ August, 2022. Discovery and co-production workshops will be held between August and November 2022.

Phases 1 and 2 of this study will result in a prototype intervention which we will put through user testing. Our user testing protocol will be prepared when we have a better understanding of what our intervention may look like. We plan to recruit 10 further child-parent/guardian dyads for this phase who will complete data regarding symptom severity and anxiety and mood will be collected at baseline and at 8 weeks. Participants will also be asked to complete a reflection diary as they use the intervention and report on acceptability, accessibility, and impact.

Once the intervention has been tested and refined through phase 4, we plan to conduct further testing in a feasibility and then a fully powered trial. We hope that the designed intervention will meet the needs of our intended population whilst encouraging these children to participate in nature-based activities. The intervention will be intended to address some of the inequalities outlined in the beginning of this paper, whilst also producing the first green social prescribing intervention designed with and for children with ADHD, that we are aware of. The intervention will be designed to be situated within services and/or in the community as shall be seen as appropriate by our co-production team of families, professionals, and researchers. We intend to share the progress of this co-production study, our findings regarding how those who experience ADHD experience nature, and the produced intervention once this study is completed.

## Dissemination

Presentations of study findings will be taken to relevant research conferences, local research symposia and seminars for CAMHS, and child health and educational professionals. In addition, our PPI lead and further appropriate PPI and organisation members will be consultees in the development of dissemination strategy which will reach families of children with ADHD. Additionally, we will produce a lay summary of the study results and the designed intervention that can be distributed to all study participants as well as relevant interest groups via social media and networks like schools and Local Authorities. We aim to utilise our child-friendly visual notes created at the sessions by our graphic designer to aid dissemination to lay audiences. We will publish findings in appropriate formats on relevant websites such as the Child Oriented Mental health Innovation Collaborative and the University of York research websites and other child mental health websites, and in academic journals. We will also collaborate with our partners (The ADHD Foundation and Yorkshire Wildlife Trust) to hold dissemination events with relevant audiences.

## Supporting information

S1 File Confirmation of ethics approval. Confirmation of ethical approval by the University of York.
(PDF)

S2 File Protocol. CONIFAS Co-Production Protocol V1.4 14.07.2022.
(DOCX)

S3 File NIHR outcome feedback. Official letter from the NIHR detailing recommendation of study for funding.
(PDF)

S4 File NIHR peer review. The reviews conducted by anonymous peer reviewers as part of the NIHR RfPB funding scheme.
(PDF)
