# Supplementary material for: Co-production of a nature-based intervention for children with ADHD study (CONIFAS): Protocol for co-production phases
Source: PLoS One. 2022 Sep 20;17(9):e0274375. doi: 10.1371/journal.pone.0274375 (PMC9488762; doi:10.1371/journal.pone.0274375)
Supplement: S2 File — CONIFAS Co-Production Protocol V1.4 14.07.2022. (DOCX) [file pone.0274375.s002.docx]

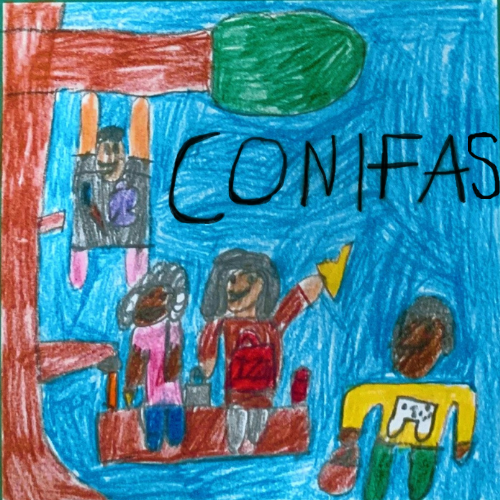


**Protocol**

**CO-production of a Nature-based Intervention For children with ADHD Study**

**(CONIFAS)**

*Co-production phases only (1, 2, & 4)*

**Chief investigators:** Dr Hannah Armitt and Dr Peter Coventry

**Principal Investigator:** Professor Piran White

**Sponsor:** Leeds and York Partnership NHS Foundation Trust

**Funder:** NIHR (National Institute for Health and Social Care Research)

**Sponsor reference:** 2022/149482/P

**IRAS ID:** 315214

**Funder reference:** NIHR203043

**Trial registration:** ISRCTN11763460

**Protocol version number:** 1.4

**Table of Contents**

[Trial Contacts 5](#_Toc108702747)

[Study Summary 6](#_Toc108702748)

[Abbreviation List 7](#_Toc108702749)

[Study Flow Chart 8](#_Toc108702750)

[1. Background 9](#_Toc108702751)

[1.1 ADHD 9](#_Toc108702752)

[1.2 Current treatment for ADHD 9](#_Toc108702753)

[1.3 Nature-based interventions 10](#_Toc108702754)

[1.4 Study Summary 10](#_Toc108702755)

[2. Rationale 10](#_Toc108702756)

[3. Objectives 11](#_Toc108702757)

[4. Study Design and Setting 11](#_Toc108702758)

[5. Participants and Eligibility Criteria 12](#_Toc108702759)

[6. Study Procedures 12](#_Toc108702760)

[6.1 Expression of Interest Procedure 12](#_Toc108702761)

[6.2 Recruitment 13](#_Toc108702762)

[6.3 Incentives 14](#_Toc108702763)

[6.4 Informed Consent 14](#_Toc108702764)

[6.5 Data Storage 14](#_Toc108702765)

[6.6 Withdrawals 14](#_Toc108702766)

[7. Study Activities 15](#_Toc108702767)

[7.1 Phase One - Discover 15](#_Toc108702768)

[7.2 Phase Two - Define 15](#_Toc108702769)

[7.3 Phase 3 - Develop 15](#_Toc108702770)

[7.4 Phase 4 - Deliver 15](#_Toc108702771)

[7.5 Missed Activities and Participant Contact 16](#_Toc108702772)

[8. Sample and Data 16](#_Toc108702773)

[8.1 Sample Size Calculation 16](#_Toc108702774)

[8.2 Data Analysis 16](#_Toc108702775)

[8.3 Knowledge Production 16](#_Toc108702776)

[9. Safety and Risk 17](#_Toc108702777)

[9.1 Assessment and Management of Risk 17](#_Toc108702778)

[9.2 Adverse Events 17](#_Toc108702779)

[9.3 Collecting, Recording, and Reporting of Adverse Events 18](#_Toc108702780)

[10. Study Management 18](#_Toc108702781)

[11. Definition of End of Study 18](#_Toc108702782)

[12. Ethical Review 18](#_Toc108702783)

[12.1 Peer review 18](#_Toc108702784)

[12.2 Patient and public involvement 18](#_Toc108702785)

[12.3 Protocol, GCP, and regulatory compliance 18](#_Toc108702786)

[12.4 Financial and competing interests 18](#_Toc108702787)

[12.5 Indemnity 19](#_Toc108702788)

[12.6 Amendments 19](#_Toc108702789)

[12.7 Post-study care 19](#_Toc108702790)

[13. Complaint Handling 19](#_Toc108702791)

[14. Dissemination 19](#_Toc108702792)

[15. References 20](#_Toc108702793)

[16. Protocol Amendment History 22](#_Toc108702794)

**Research reference numbers**

| **Governing body or organisation** | **Reference number** |
| --- | --- |
| Trial registration number | ISRCTN11763460 |
| Sponsor reference | 2022/149482/P |
| Funder reference | NIHR203043 |
| IRAS ID | 315214 |

**Funding and support in kind**

| **FUNDER(S)**  (Names and contact details of ALL organisations providing funding and/or support in kind for this trial) | **FINANCIAL AND NON FINANCIAL SUPPORT GIVEN** |
| --- | --- |
| NIHR Research for Patient Benefit | £144,857 |

# Trial Contacts

**Trial Contacts**

| **Name** | **Affiliation** | **Study role** | **Contact details** |
| --- | --- | --- | --- |
| Dr Hannah Armitt | Humber Teaching NHS Foundation Trust | Chief Investigator | [hannah.armitt@nhs.net](mailto:hannah.armitt@nhs.net) |
| Dr Peter Coventry | University of York | Chief Investigator | [peter.coventry@york.ac.uk](mailto:peter.coventry@york.ac.uk)  01904321528 |
| Professor Piran White | University of York | Co-investigator | [piran.white@york.ac.uk](mailto:piran.white@york.ac.uk) |
| Ellen Kingsley | Leeds and York Partnership NHS Foundation Trust | Trial manager | [e.kingsley@nhs.net](mailto:e.kingsley@nhs.net)  07870992859 |
| Leah Attwell | Leeds and York Partnership NHS Foundation Trust | Research assistant | [leah.attwell@nhs.net](mailto:leah.attwell@nhs.net)  07790892045 |
| Sinead Audsley | Leeds and York Partnership NHS Foundation Trust | Sponsor Contact | sinead.audsley@nhs.net  07779416847 |

**Co-applicants**

| **Name** | **Affiliation** | **Study role** | **Contact details** |
| --- | --- | --- | --- |
| Megan Garside | Leeds and York Partnership NHS Foundation Trust | LYPFT oversight | megan.garside@nhs.net |
| Andrew Steele | Yorkshire Wildlife Trust | Co-investigator | andrew.steele@ywt.org.uk |
| Kat Woolley | Yorkshire Wildlife Trust | Co-investigator | kat.woolley@ywt.org.uk |
| Michael Hussey | ADHD Foundation Limited | Co-investigator | mike.hussey@adhdfoundation.org.uk |
| Natasha Green | Community Member; Parent of child with ADHD | PPI Lead | natasha.green5@nhs.net |

# Study Summary

| **Study Title** | CO-production of a Nature-based Intervention For children with ADHD Study - Co-production Phases |
| --- | --- |
| **Internal ref. no. (or short title)** | CONIFAS |
| **Study Design** | Co-production of an intervention |
| **Study Participants** | Children and young people with ADHD, their parent/guardian, and professionals |
| **Planned Sample Size** | 30 (10 child-parent/guardian dyads, 10 professionals) |
| **Intervention duration** | N/A |
| **Follow-up duration** | N/A |
| **Planned Study Period** | 18 months |
| **Objectives** | 1. Recruit a co-production team of children and families with lived experience of ADHD, voluntary organisations working in green spaces, NHS professionals, clinicians and researchers. Define the problem and discuss ways to address it. (Phase 1)  2. Use aspects of existing campaigns (such as the five ‘Ways to Wellbeing’ and Wildlife Trust’s 30 Days Wild) to produce a new intervention that is appropriate and acceptable for this population through co-production workshops (Phase 2)  3. Conduct a separate, user testing study to refine the intervention, measuring outcomes in terms of symptoms being more manageable as well as acceptability and usability (Phase 3) – *separate protocol*  4. Refine the intervention from the previous phases (Phase 4) |
| **Method of delivery** | Workshops |

**Key words:** ADHD; intervention; nature; outdoors; child; adolescent; co-production; social prescribing

# Abbreviation List

ADHD: Attention Deficit Hyperactivity Disorder

AE: Adverse Event

CAMHS: Child and Adolescent Mental Health Services

CI: Chief Investigator

CONIFAS: CO-production of a Nature-based Intervention For children with ADHD Study

DSM-IV: Diagnostic and Statistical Manual of Mental Disorders

EOI: Expression Of Interest

GP: General Practitioner

ICD-10: International Classification of Diseases

LYPFT: Leeds and York Partnership NHS Foundation Trust

NHS: National Health Service

NICE: National Institute for Health and Care Excellence

NIHR: National Institute for Health Research

OMG: Operational Management Group

PIS: Participant Information Sheet

PPI: Patient and Public Involvement

RA: Research Assistant

REC: Research Ethics Committee

RfPB: Research for Patient Benefit

SAE: Serious Adverse Event

SENCO: Special Educational Needs Co-Ordinator

SMG: Study Management Group

TC: Trial Coordinator

UK: United Kingdom

YWT: Yorkshire Wildlife Trust

# Study Flow Chart

**
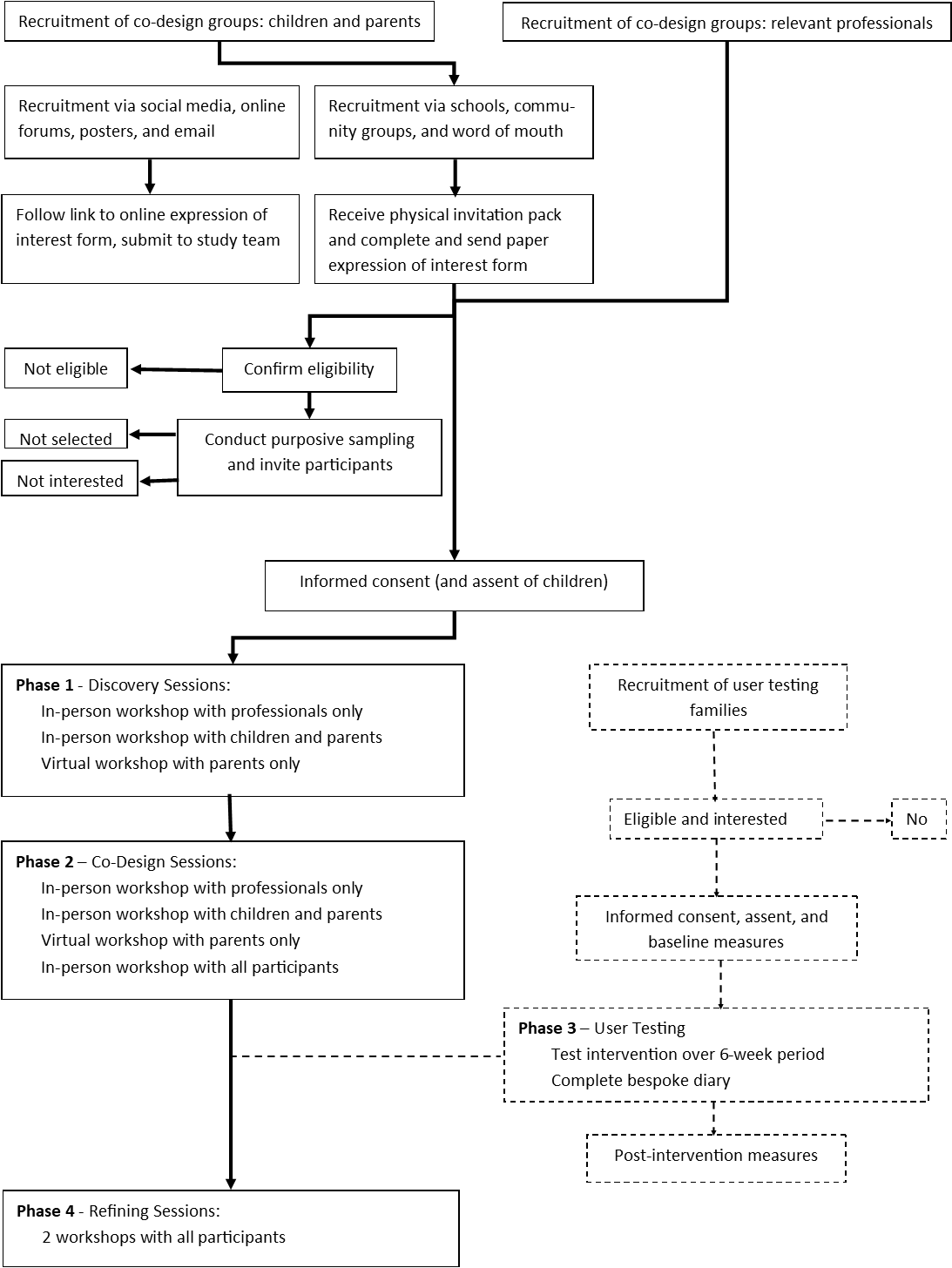
**

**Study Protocol**

**CO-production of a Nature-based Intervention For children with ADHD Study**

# Background

## ADHD

Attention deficit hyperactivity disorder (ADHD) is a neurodevelopmental condition characterised by inattention, hyperactivity and impulsivity. It is estimated to affect 2% of the adult population in the UK and between 3-5% of children in the UK (NICE 2018). For children with ADHD, these symptoms can have a significant impact on their daily functioning in the form of difficulty concentrating and being over-active regardless of setting. Children with ADHD often have comorbid mental health conditions (e.g. autism, speech and language difficulties, anxiety, low mood), poorer social and emotional wellbeing, and face challenges in academic, relational, and behavioural domains (Vibert, 2018). There is an association between low socio-economic status and an increased likelihood of ADHD diagnosis (Russel et al, 2016), indicating inequalities across the population.

## Current treatment for ADHD

Children with a diagnosis of ADHD often have poorer outcomes than their typically developing (TD) peers and increased likelihood of educational difficulties, relationship breakdowns, and the development of co-morbid mental health difficulties (Vibert, 2018). Early identification and support can prevent the development of further mental health conditions in ADHD and increase quality of life. Current health care provision, however, particularly mental health care, has been shown to not meet needs (Children’s Commissioner, 2018). It is also often focused on medication, which carries risks and does not always have good treatment adherence (Mattingley et al, 2017).

Studies report that at least 50% of referrals to child mental health clinics are for assessment and treatment of ADHD (Salomone et al., 2015), but there are long waiting lists for treatment access through child and adolescent mental health services (CAMHS). It was found that 6% of families wait over 12 weeks for a first appointment with CAMHS and 48% of families have their referrals closed before treatment is offered (NHS Digital, 2019). The estimated UK annual healthcare (NHS, social care and education) resource costs associated with treating ADHD in adolescents has been reported as £670 million, equating to a mean cost per adolescent of £5493 (Telford et al, 2013). It is also reported that at time of diagnosis, families do not routinely receive appropriate intervention (Children’s Commissioning Report, 2018), which can lead to deterioration in the child’s wellbeing.

Children and young people are negatively impacted by wait times and limited access to intervention, but there is also a negative impact on their parents or guardians and caregivers. The Caregiver Perspective on Paediatric ADHD study reported 38% of caregivers (n=2872) had been late for work in the past month due to their child’s ADHD, and that 31% of caregivers (n=3688) had altered their employment status (Flood et al, 2016). Telford et al (2013) identify the need to develop and evaluate early interventions which have the potential to reduce the longer-term burden of ADHD.

## Nature-based interventions

There is consistent evidence that engagement with nature is beneficial for all children and young people in terms of physical activity levels, health outcomes, increases in wellbeing, reduced stress, and developing a positive affinity with nature (Sheldrake et al, 2019). There is evidence to suggest that engaging in outdoor activity, even when the weather is colder, can be beneficial to development and mental and physical health (Mutz & Müller, 2016). The potential of preventative nature-based interventions is becoming increasingly recognised, with recommendations for increases in the public health budget for programs promoting access to green space (Public Health England, 2020). Current government policy aims to increase access to green spaces to improve mental and physical health (Public Health England, 2020).

For children with ADHD, increased exposure to green spaces was strongly associated with reduced ADHD symptoms (Donovan et al, 2019), reduced need for medication, and symptom reduction (Tillman et al, 2018). Green/blue spaces help alleviate ADHD symptoms via the availability of space and the associated benefits of physical activity to ‘burn off’ excess energy (Ulrich et al, 1991). Natural spaces also provide a multi-sensory space, removing external distractions of modern life such as technology, which counteracts ADHD symptoms that make it difficult to stay focussed (Taylor et al, 2001). Additionally, the relaxing effects of natural spaces can impact on a number of behavioural difficulties seen in ADHD (Van den Berg, 2011).

The New Economics Foundation (2008) has developed the five ‘Ways to Wellbeing’ (Connect, Be Active, Take Notice, Learn and Give), an evidence-based framework using structured natural activities to overcome inequalities of access and improve mental health. Although this is promising and aligns with recent recommendations (Public Health England, 2020), as yet there are no well evidenced and tested green interventions for use in the target population or for use in the NHS in Child and Adolescent Mental Health services (CAMHS).

## Study Summary

This study will use a co-production methodology to create a nature-based intervention for children and young people with ADHD and their families to help manage the impact of ADHD symptoms on their lives. Four phases will be used based on the Design Council’s Double Diamond model (Design Council, 2017): Discover, Define, Develop, and Deliver. These phases will map on to four phases of the research study: Phase 1 involves discovery workshops to understand the problem (objective 1), phase 2 involves co-production workshops to define and develop the intervention (objective 2), phase 3 involves user testing to deliver the intervention (objective 3 – *separate protocol*) and phase 4 includes refining the intervention based on phase 3 outcomes (objective 4).

# Rationale

Current NICE (2018) guidelines for children with ADHD recognise the need for a healthy lifestyle through physical activity and social connectedness. Nature-based interventions would align with this through their multiple benefits including opportunities for physical activity, increases in wellbeing and health outcomes, reduced stress, and connectedness with nature (Sheldrake et al, 2019). Although evidence shows that nature-based interventions can be highly effective at supporting children with ADHD, there is no bespoke intervention that has been developed and rigorously tested in this population.

This study intends to create a novel, co-produced intervention for use with children with ADHD to reduce the impact of symptoms and make them more manageable in day to day life. The intervention can be situated in the NHS with Child and Adolescent Mental Health services (CAMHS) well placed to support the development of the intervention for use with this patient population. We will work closely with them throughout the study and have included clinical staff in initial discussions during proposal development. This will ensure links between the end users (families of children with ADHD) and local mental health services are created. Additionally, links between a local nature partner (Yorkshire Wildlife Trust), The University of York, and the ADHD Foundation will be made which will add value to current practice and knowledge.

Co-production has been chosen as the methodology as its central value is the development of more equal partnerships between people who use services, carers and professionals. It is hoped co-production will support development of an intervention that is meaningful for the population using the service. Co-production has been linked with better outcomes for people who use services and can support the development of stronger relationships by forging strong links with service providers.

# Objectives

1. Recruit a co-production team of children and families with lived experience of ADHD, voluntary organisations working in green spaces, NHS professionals, clinicians and researchers. Investigate the strengths and difficulties associated with an ADHD diagnosis in children and how nature can be used to support them (Phase 1).
2. Design an intervention that is appropriate and acceptable for this population through discovery and co-production workshops, using existing campaigns (such as the five ‘Ways to Wellbeing’ and Wildlife Trust’s 30 Days Wild) for inspiration (Phase 2).
3. Conduct a separate, user-testing phase to test the usability, acceptability, and accessibility of the created intervention (phase 3) – *separate protocol*.
4. Refine the intervention from the previous phases (Phase 4).

# Study Design and Setting

The present study of objectives 1, 2, and 4 will use co-production methodology, using the four phases of the Design Council’s Double Diamond model (Design Council, 2017) which map onto the study objectives as listed above. In the first phase, the team will 'discover' what the challenges are for those with ADHD and how nature-based interventions may support them by creating a co-production team of children and families with lived experience of ADHD and professionals from voluntary organisations and the NHS. In phase two, the 'define' phase, the team will seek to create an intervention using elements from existing campaigns (e.g. Ways to Wellbeing) which meets the needs identified by the co-production team in the discovery phase. The third phase (‘develop’) will test the intervention with children and families with lived experience of ADHD to allow for further development arising from the feedback collected via outcome measures which focuses on symptom manageability. This phase will also assess the acceptability and useability of the created intervention. A separate protocol and ethics submission will be submitted to cover the activities of phase 3 at a later date. In phase four ('deliver'), the final product is created ready for a wider launch (feasibility). Practical co-production resources from The Institute for Research and Innovation in Social Services (Vallely, 2018), will be used at each phase. These resources include the 'thinking hats' tool which enables group members to consider the challenge from different perspectives and a pathway tool which will support the team to keep track of the intervention’s development.

# Participants and Eligibility Criteria

Participants are children and young people aged between 5-11 years with lived experience of ADHD, their parent/guardian, and professionals from voluntary agencies or the NHS. All participants must have sufficient understanding of spoken English to participate in the co-production events. All participants will live or work in the local area and be able to travel to the designated meeting location; Barlow Common, Selby, North Yorkshire, YO8 8EZ. These areas may include York, East Riding of Yorkshire, West Yorkshire, North Yorkshire, and South Yorkshire.

Children who pose a risk of harm to themselves or others and children will not be eligible to take part. This will be checked during the Expression of Interest procedure and confirmed when. Children with comorbid diagnoses (e.g. autism, physical difficulties) will be supported to participate with recognition that additional considerations in accessing outside spaces may arise. Any relevant risks will be assessed during the consent procedure.

# Study Procedures

## Expression of Interest Procedure

As this study involves the creation of a new intervention by community members, the research team feel the intervention should meet the needs of a range of families. Thus, the team have created a process for recruiting families that represent a diverse range of life experiences via purposive sampling. An expression of interest (EOI) procedure will be used in which interested families will complete a short demographic questionnaire including questions on their family structure and locality. The intention of this procedure is to capture the needs of children across the specified age range, different cultures, large and small families, parent education levels, and location types (urban, rural, and coastal locations).

The study will be advertised through social media - including relevant parenting forums - through posters, via local council advertisements, through schools, and via community groups. Parents/guardians will be invited to complete the EOI survey to register their interest through an online survey which can be circulated via email and through QR codes on study posters. All circulation methods will include the study team’s contact details. Families who contact the research team but cannot access the online form will be given the option of completing the survey over the phone or via post. All eligible families completing the EOI form will be given a copy of the parent/guardian participant information sheet (PIS) and the child PIS which contains study information tailored to children aged 5-11. Physical ‘parent packs’ with the study advertisement poster, a parent invitation letter, a PIS, and a physical copy of the EOI form will also be created and distributed to community groups and institutions to pass on to relevant families.

The EOI survey will close on an agreed upon date. All parents/guardians completing the form will be made aware that expressing interest does not guarantee that they will be invited to take part due to the use of purposive sampling. This will be thoroughly explained to parents/guardians prior to their completion of the survey. They will also have the option to provide consent for the research team to store their details for contact about participating should other families drop out (demographic information for these families will also be retained so that we can aim to uphold diversity within our sample), and for the user testing phase of the study (phase 3), though this will be entirely optional. If they agree, these details will be stored on a password protected spreadsheet on secure drives on NHS computers for the duration of the study. See section 6.4 for further details of data storage and destruction.

## Recruitment

Children and their parents/guardians will be recruited via purposive sampling from the EOIs received. 10 child-parent/guardian dyads will be selected based on their diverse and representative life experiences, as we will aim to recruit those with varied experiences based on their answers to the survey. Our selection process will be transparent from the beginning, and all OMG members will be present during this selection. Participant selection will be reported to the SMG for approval.

All parents/carers completing the EOI survey will have the option to provide consent for us to retain only their contact details to be contacted about the user testing phase (phase 3) of the study, or they can provide consent for their contact details and demographic information to be retained for the duration of the study to be placed on a ‘reserves’ list in the case that recruited families withdraw. See section 6.5 for further details on this process. Families that are not selected will be informed of this but will not receive any further contact from the study team unless they have consented to the study team’s retention of their contact details.

If selected, the families will be encouraged to re-read the PIS and to discuss the child PIS with their child. They will have a discussion with the research team about the study and be able to ask any questions. If they would like to proceed, parents/guardians will complete a consent form which provides consent for their and their child’s participation in the study. The children can complete an assent form (to show their agreement to participate) if deemed appropriate, though this is not necessary as parental consent on their behalf is sufficient. However, if any parents/guardians wish to participate but their child does not, they will not be enrolled in the study. If any of the selected 10 families no longer wish to take part at the consent stage, the study team will use the other received EOIs and select new potential participants, again based on representativeness.

If we do not receive a substantial number of completed EOIs by an agreed-upon date and are unlikely to meet our recruitment target, the study team will cease the use of this tool for recruitment purposes. Eligible families will be recruited by approaching the study team directly and will be asked to complete the EOI for the purposes of consent to retain contact details rather than for collection of demographic details.

The 10 professionals will not be required to complete an EOI procedure. We aim to recruit a mixture of medical, educational, and outdoor-activity professionals. This may include, but is not limited to, NHS CAMHS workers, school special educational needs coordinators (SENCOs), charity staff, and outdoor-activity staff. Professionals will be recruited from northern and regional CAMHS, third sector, and education settings via word of mouth, email, and telephone contact utilising existing links. A participant information sheet will be provided and the study will be explained in detail with opportunity for any questions. Informed consent to participate will be then obtained.

Given that recruited participants will be required to attend all study workshops to ensure continuity of input, should there be sufficient potential participants, the study team will over-recruit by a small number in case some participants aren’t able to attend sessions at short notice.

## Incentives

Participating families will receive a £20 shopping voucher (e.g. Love2Shop) for every in-person workshop they attend. They are asked to attend five in-person workshops, and thus can receive up to £100 worth of shopping vouchers. All participants will have lunch provided on the day and their travel costs will be reimbursed. Families will also receive a ‘goodie bag’ (e.g. stickers, pencil, small magnifying glass for insect observation) for children to take home after each in-person workshop.

## Informed Consent

Participation in the study will be entirely voluntary for all participant types (children, parents/guardians, and professionals) and written informed consent will be obtained before workshop participation. Participants under the age of 16 will be invited to complete an age-appropriate assent form, but this will not be required (the research team will defer to parents/guardians for the appropriateness of this). Parent/guardian consent will cover their own participation and that of their child. Where a child declines to participate they will not be included.

## Data Storage

Data provided by participating families during the EOI procedure will be retained for the duration of the study. For those who consent to being on the reserves list, their data will be retained for the duration of the study and stored separately from participating families. For those who consent to being contacted about the User Testing phase of the study, we will retain their contact details until recruitment for this phase is complete. This data will be used for intended purposes only and will not be shared.

Identifiable details of consenting participants including name, address, and contact details will be entered onto a password-protected spreadsheet and stored on a secure NHS drive accessible only through secure logins on NHS computers. Demographic information gathered will also be stored securely and will be stored separately and be pseudonymised through the use of participant codes. Physical consent forms will be securely stored in locked filing cabinets on LYPFT NHS premises.

Participant’s personal details will be retained for 6 months after the end of the study and then destroyed. This 6-month period will allow for dissemination of findings. Research data will be retained for 5 years as per the Sponsor’s (Leeds and York Partnership Foundation NHS Trust) regulations.

## Withdrawals

Any participant can withdraw from the study at any time without having to provide a reason for withdrawal. If a parent or guardian wishes to withdraw from the study, their child will also be withdrawn if a replacement parent/carer is not available. If a child wishes to withdraw, it will be clarified whether their parent wishes to continue participating.

When a dyad withdraws we will aim to replace them with other families who completed the EOI procedure who also provided consent for us to contact them in such an event. We will aim to uphold the diversity of our cohort in the selection of these reserves, and any selection of participants will be conducted in the presence of the full OMG and will be confirmed by the SMG. However, depending on the stage of the study, we may not always replace a withdrawn dyad. This will be judged on a case-by-case basis.

# Study Activities

At the point of recruitment we will disseminate the ADHD Hero Activity Book (The University of Sheffield, 2021) to families as a supplementary source of positively-framed information on the impacts of ADHD. Families will not be required to complete this and this will not be collected by the research team. We aim to use this tool as a piece of psychoeducation before families attend the workshops. We will also utilise the Iriss Inclusion Checklist (Iriss 2018) for families to complete and provide before the sessions to ensure accessibility for them.

All workshops will be attended by a graphic designer who will create visual notes from each workshop which will then be shared with subsequent workshops. An alternate activity will be offered in cases where families need to bring participating children’s siblings to the workshops. Lunch will be provided for all participants at each in-person workshop and travel expenses will be reimbursed.

The CONIFAS study design is informed by and mapped onto the Design Council’s Double-Diamond model of co-production (Design Council, 2017). As such it is split into four phases.

## 7.1 Phase One - Discover

The discovery phase aims to understand and define the problem at hand, the potential methods of change, any barriers to change, and which nature-based activities may be helpful for children with ADHD. This will occur over three separate workshops:

1. An in-person, half-day event with relevant professionals who work with children with ADHD.
2. An in-person, half-day event with children and their parent/guardian to gather children’s views and participate in some preliminary nature-based activities.
3. An online workshop with just parents/guardians to gather parent/guardian views.

The workshops will be led by the research team and activities will be facilitated by the Yorkshire Wildlife Trust staff.

## 7.2 Phase Two - Define

This phase will focus on the development of the intervention ready for user testing. This will occur across four workshops:

1. An in-person, half-day event with professionals.
2. An in-person, half-day event with children and parents/guardians.
3. An online workshop with parents/guardians.
4. An in-person, half-day event with all participants.

The workshops will be led by the research team and activities will be facilitated by the Yorkshire Wildlife Trust staff.

## 7.3 Phase 3 - Develop

This phase will involve recruiting 10 new child and parent/guardian dyads to conduct user testing of the designed intervention. This phase will be covered in a future ethics application once details of the co-produced intervention are available and will include a new protocol and new study documents.

## 7.4 Phase 4 - Deliver

In the final phase, all participants from phases 1 and 2 will attend two workshops to look at the results of the user testing phase, to make any changes to the intervention, and to finalise the intervention and any intervention materials.

## 7.5 Missed Activities and Participant Contact

In the event that a participant misses a workshop but wishes to continue in the study, they will be offered a phone/video call with a member of the research team to go through the summary of the day and to add any of their thoughts, experiences, or ideas. We will greatly encourage participants to attend the workshops, however, as we feel these will be pertinent to ensuring good quality outcomes and team work.

We will also agree with each participant group (parent/child dyads and professionals) how they may like to keep in contact with the research team in-between sessions, should they like to. This may include emailing, the use of online knowledge-sharing tools (e.g. Google Jamboard), Whatsapp groups, etc. with the purpose of sharing any relevant information, experiences, or ideas in the meantime. These reflections will be discussed at the next available workshops. We will encourage direct contact with the research team only and avoid any participants feeling pressured to share their contact details with each other. No participant contact details will be shared by the research team.

# Sample and Data

## Sample Size Calculation

This study uses qualitative co-production methods and no formal sample size calculation is required. This protocol covers the first, second, and fourth phases of the CONIFAS study during which co-production of the intervention will take place. Three separate groups will be formed for this. These will be one group of 10 children, one parent/guardian for each child, and one group of 10 professionals, totalling 30 participants. Group size is based on guidance for co-production methodology that allows for varied views but equal participation (Vallely, 2018).

## Data Analysis

Only demographic data will be collected in phases 1, 2, and 4 of this study. This data will be reviewed descriptively for the purposes of participant selection.

## Knowledge Production

This study will produce qualitative information to support the co-production of a nature-based intervention for children with ADHD. We will gather observational data along with written contributions from participants. These will be collated and synthesised as part of the co-production exercise by a graphic designer. The graphic designer will attend all sessions and will provide live, visual notes of the discussions being had. These notes will be continuously shared with participants and used to draw our conclusions. The same graphic designer will also help to design the physical intervention following co-production. A signed agreement will be in place to protect the intellectual property arising from this study, and the graphic designer will have an up-to-date DBS check. In addition this study will generate insight into the process of co-producing nature-based interventions for children with ADHD and has the potential to contribute methodological knowledge relevant to research teams working in cognate fields.

# Safety and Risk

## 9.1 Assessment and Management of Risk

We do not anticipate that participants will be subject to any substantial risks during this study. However, the focus of this study is on nature-based and outdoor activities, with workshop sessions involving participation in these activities and being outdoors (weather dependant). As such, usual risks associated with being outdoors may be expected including but not limited to slips, trips, sunburn, insect bites, stings, etc. All children will be attending with a parent/guardian or carer, and it will be deemed parent/guardian responsibility to monitor the children. All participants will be reminded of the risks, encouraged to proceed with caution, notified of any particular risk areas (slippery ground, etc.) and reminded to attend sessions wearing appropriate footwear and clothing with plenty of notice. Where needed, additional clothing, particularly for cold or wet weather, will be provided by YWT on site. A risk assessment of the YWT site and the planned activities will be conducted.

Participants will be asked to complete a risk management form where they can report on relevant risks such as asthma, bee sting allergies, etc. Participants will be reminded before each session to bring any relevant medication including inhalers and emergency medication. It will be the responsibility of the participants (or their attending parents) to manage and administer their own medications.

Lunch will be provided for participants when they attend an in-person session. All participants and attending staff members will be asked to report any allergies and intolerances to the study team, and these will be catered for accordingly.

The research team recognise that children with ADHD may struggle with listening to and retaining instructions due to the nature of their disorder. The researchers will ensure that instructions, particularly pertaining to any risks, are clearly communicated and that parents/guardians, carers, and staff are attentive to the children.

## 9.2 Adverse Events

Possible harm as a result of the study is expected to be minimal but will be monitored and recorded. An adverse event (AE) in this study may include outdoor activity-based risks or behavioural incidents including:

- Slips, trips, and falls
- Insect bites and stings
- Bumps and scrapes
- Significant emotional distress
- Verbal abuse
- Physical violence

All AEs will be assessed for seriousness and will be recorded as serious adverse events (SAEs) if they:

- Result in death
- Are life-threatening
- Require hospitalisation or prolongation of existing hospitalisation
- Result in persistent or significant disability or incapacity

## 9.3 Collecting, Recording, and Reporting of Adverse Events

AEs that are considered related to participation in this study, and all SAEs, will be reported to the CIs. SAEs considered to be related to the study and to be unexpected will be reported to the Sponsor and Study Management Group (SMG) as soon as possible. The Operational Management Group (OMG) will regularly assess any arising AEs and the SMG will review all AEs during scheduled meetings and propose any actions accordingly.

# Study Management

The day-to-day running of the study will be managed by the Operational Management Group (OMG): Hannah Armitt, Peter Coventry (co-CIs), Ellen Kingsley (TC), and Leah Attwell (RA). The OMG will meet on a monthly basis with communication in between meetings and will discuss and monitor the day-to -day running of the trial. The Study Management Group (SMG) involves the OMG and all co-applicants including the PI: Piran White (PI), Megan Garside (LYPFT), Kat Woolley (YWT), Mike Hussey (ADHD Foundation), and Natasha Green (PPI Lead). The SMG will meet every 3 months to provide additional oversight, guidance, and decision making.

# Definition of End of Study

The study will end on the 31st of August 2023.

# Ethical Review

The proposed study will be conducted in accordance with ICH Good Clinical Practice guidelines. This project does not require HRA approval in accordance with their guidance. The study was submitted for ethical review by the University of York department of environment and geography research ethics committee (REC) and approval was gained on the 1^st^ June, 2022.

## 12.1 Peer review

The protocol for this study has been previously peer reviewed in line with the National Institute for Health Research (NIHR) Research for Patient Benefit (RfPB) funding process.

## 12.2 Patient and public involvement

The research team is committed to involving PPI in all parts of the study. A parent of a child with ADHD is a co-applicant on the study and will review all participant facing documents. Participant facing documents will also be reviewed by the sponsor’s (LYPFT NHS) research patient ambassador. The nature-based intervention itself will be co-produced with families with lived experience of ADHD and with professionals who work with them. This intervention will then be tested by families with lived experience of ADHD.

## 12.3 Protocol, GCP, and regulatory compliance

Non-compliance with GCP and the study protocol will be monitored and recorded by the study team in accordance with LYPFT SOPs.

## 12.4 Financial and competing interests

There are no financial or competing interests to report. The Yorkshire Wildlife Trust property on which the in-person workshops will be held is free to enter, provides free parking, and has free amenities. Lunch will be paid for and provided by the research team and so no funds from participants attending the group sessions will be required.

Advice and guidance will be sought from Medipex in the creation of the intervention. The research team do not intend to capitalise upon the designed intervention and wish for it to be publicly owned.

## 12.5 Indemnity

To meet the potential legal liability for harm to participants arising from the design, conduct, and management of the research, NHS employees will be covered by NHS indemnity and University employees will be covered by their institution’s insurance. Workshops will be held on Yorkshire Wildlife Trust premises, and relevant staff will be covered by their organisation’s indemnity insurance. As the sessions will be led and designed by NHS staff, study participants will be covered by NHS indemnity insurance.

## 12.6 Amendments

All study amendments will be approved by the co-CIs and all substantial amendments will be approved by the CIs, the Sponsor, and the SMG prior to submission for ethical approval. Amendment history will be tracked by adopting version control and via an amendment log.

## 12.7 Post-study care

This study will not affect any treatment or support from schools, GPs, and/or community services received by participating children with ADHD, they will continue to receive this throughout. Should any additional needs be identified through the trial, advice for contacting support services will be provided by the research team. This may include advice about voluntary agencies, parent support groups, local authority support, health and disability teams within social care, and CAMHS.

# Complaint Handling

The PIS will provide participants with contact details of the CIs, REC chair, and Sponsor in case of complaint.

# Dissemination

The research team has a strong track record of successful dissemination of work funded by the NIHR and other funding bodies. We will begin to consider our dissemination strategy at an early stage of the project. We will also aim to publish the results of our study in a scientific journal.

Presentations of study findings will be taken to relevant research conferences, local research symposia and seminars for CAMHS, and child health and educational professionals. In addition, our PPI lead and further appropriate PPI and organisation members will be consultees in the development of dissemination strategy which will be effective in reaching families of children with ADHD. Additionally, we will produce a short summary of the study results and the designed intervention that can be distributed to all study participants as well as relevant interest groups. We will publish findings on relevant websites such as the University and child mental health websites.

# References

The Children’s Commissioner (2018). Children’s Mental Health Briefing. [Accessed 15 December 2020]. Available: //[www.childrenscommissioner.gov.uk/wp-content/uploads/2019/02/childrens-mental-health-briefing-nov-2018.pdf](http://www.childrenscommissioner.gov.uk/wp-content/uploads/2019/02/childrens-mental-health-briefing-nov-2018.pdf)

Design Council (2017). The design process: what is the Double Diamond? [Accessed January 2021]. Available <http://www.designcouncil.org.uk/newsopinion/design-process-what-doublediamond>

Donovan, G. H., Michael, Y. L., Gatziolis, D., Mannetje, A., & Douwes, J. (2019). Association between exposure to the natural environment, rurality, and attention-deficit hyperactivity disorder in children in New Zealand: a linkage study. *The Lancet Planetary Health,* 3(5), 226-234.

Flood, E., Gajiria, K., Sikirica, V., Dietrich, C. D., Romero, B., Harpin, V., Banaschewski, T., Quintero, J., & Erder, H., (2016). The Caregiver Perspective on Paediatric ADHD (CAPPA) survey: understanding sociodemographic and clinical characteristics, treatment use, and impact of ADHD in Europe. *Journal of Affective Disorders,* 200, 222-234.

Mattingley, G. W., Wilson, J., & Rostain, A. L. (2017). A clinician’s guide to ADHD treatment options. *Postgraduate Medicine,* 129(7), 657-666).

Mutz, M., & Muller, J., (2016). Mental health benefits of outdoor adventures: results from two pilot studies. *Journal of Adolescence,* 49, 105-114.

National Institute for Health and Care Excellence (2018). Attention deficit hyperactivity disorder: diagnosis and management [NICE Guideline No. 87].

New Economics Foundation (2008). Five Ways to Wellbeing. *NEF.*

NHS Digital (2019)/ Waiting times for children and young people’s mental health services, 2018-2019 additional statistics. *NHS Digital.*

Public Health England. (2020). Improving Access to Green Space: A New Review for 2020. *Crown Publishing.*

Russell, A. E., Williams, R., & Russell, G. (2016). The association between socioeconomic disadvantage and Attention Deficit/Hyperactivity Disorder (ADHD): A systematic review. *Journal of Child Psychiatry and Human Development,* 47(3), 440-458.

Salomone, S., Fleming, G. R., Shanahan, J. M., Castorina, M., Bramham, J., Connell, R. G., & Robertson I. H. (2015). The effects of a Self-Alert Training (SAT) program in adults with ADHD. *Frontiers in Human Neuroscience*, 9.

Sheldrake, R., Amos, R., Reiss, M. J., & UCL & Wildlife Trusts, (2019). Children and Nature: A Research Evaluation. *UCL.*

Taylor, A. F., Kuo, F. E., & Sullivan, W. C. (2001). Coping with ADD: the surprising connection to green play settings. *Environment and Behaviour,* 33, 54-77.

Telford, C., Green, C., Logan, S., Langley, K., Thapar, A., & Ford, T. (2013). Estimating the costs of ongoing care for adolescents with attention-deficit hyperactivity disorder. *Social Psychiatry and Psychiatric Epidemiology,* 48, 337-344.

Tillman, S., Tobin, D., Avison, W., & Gilliland, J., (2018). Mental health benefits of interaction with nature in children and teenagers: a systematic review. *Journal of Epidemiology and Community Health,* 72(10), 958-966.

Ulrich, R. S., Simons, R. F., Losito, B. D., Fiorito, E., Miles, M. A., & Zelson, M. (1991). Stress recovery during exposure to natural and urban environments. *Journal of Environmental Psychology,* 11, 201-230.

The University of Sheffield (2021). The ADHD Hero Activity Book. [Accessed 26th April 2022]. Available: <https://www.penninecare.nhs.uk/wellbeing/wellbeing-packs-and-activities>

Vallely, J. (2018). Co-production Project Planner. [Accessed January 2021]. Available: <https://www.iriss.org.uk/resources/tools/co-production-project-planner>

Ven den Berg, A. E., & Van den Berg, C. G., (2011). A comparison of children with ADHD in a natural and built setting. *Child Care and Health Development,* 37(3), 430-439.

Vibert, S. (2018). Your Attention Please: The Social and Economic Impact of ADHD. *DEMOS.*

# Protocol Amendment History

| **Amendment No.** | **Protocol version no.** | **Date** | **Author(s) of changes** | **Details of changes made** |
| --- | --- | --- | --- | --- |
| 1 | 1.2 | 30.05.2022 | Leah Attwell | Study name change applied and references to “co-design” corrected to “co-production” |
| 2 | 1.3 | 01.06.2022 | Ellen Kingsley | ISRCTN number and ethics approval date added. Additional references to ‘co-design’ changed to ‘co-production’ |
| 3 | 1.4 | 14.07.2022 | Leah Attwell, Ellen Kingsley | Co-applicant Sara Booth-Card replaced with Andrew Steele. Clarified contact of participants not selected from EOI, added ability to stop use of EOI procedure when recruiting families, and possibility of over-recruiting families and professionals (section 6.2). Included introduction of a graphic designer to attend workshops (section 7 & 8.3). Increased the value of vouchers that families can receive to £20 per workshop and added goodie bags for children (section 6.3). |
