# Supplementary material for: Co-production of a nature-based intervention for children with ADHD study (CONIFAS): Protocol for co-production phases
Source: PLoS One. 2022 Sep 20;17(9):e0274375. doi: 10.1371/journal.pone.0274375 (PMC9488762; doi:10.1371/journal.pone.0274375)
Supplement: S1 File — Confirmation of ethical approval by the University of York. (PDF) [file pone.0274375.s001.pdf]

Department of Environment and Geography
University of York, Wentworth Way
York YO10 5NG
york.ac.uk/environment

General Enquiries
+44 (0)1904 322999
environment@york.ac.uk

Professor Piran White
Department of Environment and Geography,
Wentworth Way,
University of York,
York YO10 5NG, UK

10 June 2022

Dear Professor White

This is formal confirmation that your ethics application for the study, "CO-design of a Nature-based Intervention For children with ADHD" was approved by the Department of Environment and Geography Ethical Review Committee on the 23 May 2022.

Yours sincerely,

Dr Colin McClean
Chair, Department of Environment and Geography Ethical Review Committee
Department of Environment and Geography, Wentworth Way, University of York, York YO10 5NG, UK
e-mail: environment-ethics@york.ac.uk
