# Supplementary material for: Co-production of a nature-based intervention for children with ADHD study (CONIFAS): Protocol for co-production phases
Source: PLoS One. 2022 Sep 20;17(9):e0274375. doi: 10.1371/journal.pone.0274375 (PMC9488762; doi:10.1371/journal.pone.0274375)
Supplement: S3 File — Official letter from the NIHR detailing recommendation of study for funding. (PDF) [file pone.0274375.s003.pdf]

NIHR | National Institute for Health Research

CENTRAL COMMISSIONING FACILITY
Grange House
15 Church Street
Twickenham
TW1 3NL

Tel: 020 8843 8000
Fax: 020 8843 8001
Email: ccf@nihr.ac.uk
www.nihr.ac.uk/ccf

Dr Hannah Armitt
By Email

Thursday 18 November 2021

Dear Dr Armitt,

Research for Patient Benefit (RfPB) Programme: NIHR203043 – Development of a nature based intervention for children with ADHD (NICA)

I am pleased to inform you that the Committee has recommended your application, submitted for consideration in Competition 44, for funding. The Department of Health and Social Care, in their capacity as the National Institute for Health Research (NIHR), has confirmed their intention to award funding upon acceptance of the terms and conditions set out in the Standard Research Contract and pending agreement to the suggested amendments recommended by the Committee, as detailed below under the 'Committee feedback' heading.

The Standard Research Contract, between Contractors and the Secretary of State for Health for all initiatives can be found on the NIHR website.

Next Steps

The NIHR is committed to the rapid initiation of research following the decision to fund to benefit patients as soon as possible. Therefore, we expect funded researchers to be working towards gaining the necessary contractual agreements and governance approvals required to start the project between **01 March 2022** and **31 August 2022** or by a date mutually agreed by both parties on acceptance of the award.

The NIHR acknowledges the risk to organisations around committing resources to research before a contract is in place; however, it is rare to not reach contractual terms unless the circumstance of the research team changes. The NIHR, therefore, encourages organisations to commit staff to setting up projects at as early an opportunity as possible in order to expedite the formal commencement of research.

It is acknowledged that there can be unforeseen delays in starting up a research project, but in order to help reduce these it is your responsibility to work closely with your organisation's R&D department or equivalent as well as other colleagues / departments involved in the administration and management of the research, and to start these discussions at the earliest opportunity.

To ensure that the project starts within the agreed timeframe with all the required agreements and approvals in place, appropriate staff (such as project and/or study managers) need to be in post as early as possible after receiving this letter of intent. These staff costs will ultimately be covered through the research funding award, but you are encouraged to meet them from Research Capability Funding (RCF) prior to the research contract being agreed.

To support the often-iterative process towards agreement of the contract, we have set out the guiding timeframes for the submission of responses or information for each step towards the agreement of the Standard Research Contract as well as the anticipated start date.

- Confirmation of acceptance of funding – no later than **02 December 2021**
- Responses to Committee feedback and queries – no later than **02 December 2021**
- Responses to Finance, Contracting and IP queries – no later than **02 December 2021**
- Contract signature – no later than **18 April 2022**
- Contracted commencement start – between **01 March 2022** and **31 August 2022** or by a date mutually agreed by both parties on acceptance of the award
- Submission of draft collaboration agreements and/or subcontracts (where applicable) – 6 months from the start of the project, or a date mutually agreed on acceptance of the award

On receipt of information as set out above, the NIHR through the Central Commissioning Facility is committed to responding to your submission of information within two weeks or we will update you on progress.

Please take the time to carefully read the enclosures to this letter which details the feedback on your application, your contact, Stuart Haylock within the Central Commissioning Facility who will be working with you on the contract, the processes to be undertaken during the next steps, as well as additional information relating to your award. Please note that all the responses to the queries listed below need to be sent directly to **Stuart Haylock** at **stuart.haylock@nihr.ac.uk.**

Yours sincerely,

Mr Ben Morgan
Assistant Director, NIHR Research for Patient Benefit Programme

Enclosures:
- Committee Feedback
- Outcomes table
- External Reviews
- Contracting and IP Queries
- RfPB Grant Holders’ Responsibilities
- Annex 1: Additional Information

cc: research.lypft@nhs.net

Committee Feedback

The Committee praised the well addressed comments from stage 1 feedback. However, there are several points on which the Committee has requested the research team's response:

- The applicants were asked to provide further detail on the future invention, in particular the dosage of the intervention lacked clarity. In addition, the Committee recommended the inclusion of further intervention development expertise on the research team or further articulate how the current team has this expertise, bearing in mind the Tier 3 funding limit of £150,000.
- The Committee noted that the applicants had made no reference to development of a logic model for their intervention or the Medical Research Council guidance for development of complex interventions. The applicants were asked to comment on this.
- Concerns were raised regarding how families from poorer backgrounds may have problems accessing winter clothing which could affect the seasonality of the intervention. The applicants were asked to comment on this. In addition, the applicants were asked to further clarify how they would ensure maximum diversity in recruitment to ensure that the intervention is as widely applicable as possible.
- The Committee requested further detail regarding how patient and public involvement (PPI) members and other stakeholders would be involved in dissemination activity. In addition the applicants were asked the clarify some PPI costings:
    - The research team refer to a "6 parent PPI Group" – is this group costed, at what rate, what input will this group have and when will they have this input.
    - PPI budget allocation appears to include costs for participants in workshops and user-testing. The applicants were asked to clarify whether these costs are PPI or qualitative research?
    - The PPI Lead's time allocation was considered insufficient. The time allocations for the PPI Lead should be reviewed and either justified or amended keeping in mind the £150,000 funding limit for RfPB Tier 3 studies.

Outcomes Table

The attached outcomes table contains the anonymised mean scores and ranking from the Competition 44 Stage 2 RfPB Committee meeting where your application was assessed. It is intended to aid you in contextualising your feedback from the Committee.

Your anonymised code is: **YNE-0921-2002**

Peer Review

Also attached are the external peer and public reviewers' comments relating to your application. Please note that external reviewer comments are always taken in the context of a wider discussion on a proposal and matters such as unduly harsh remarks, seeming misunderstanding or bias will have been taken into account by the Committee in coming to their decision.

Contracting and IP Queries

Alongside the scientific negotiations and budget discussions currently underway, you are also required to provide information which will be used to prepare the contract for this award,

including information regarding collaborators and intellectual property (IP) arrangements. Please provide a response **in a separate document** and send it directly to **Stuart Haylock** at **stuart.haylock@nihr.ac.uk** by **02 December 2021** at the latest.

In order to inform your responses to the below queries, we recommend that **you and the Contractor, (Leeds and York Partnership NHS Foundation Trust)** review the standard contract that will be used to contract the award. This is an agreement between the Secretary of State for Health and the Contractor and can be downloaded here.

Contracting Queries

1. **Confirmation of anticipated contracted start date**
   Please provide an anticipated start date between **01 March 2022** and **31 August 2022** or by a date mutually agreed by both parties on acceptance of the award. When estimating a start date, please also take into account the likelihood of any delays in staff recruitment which may hold up the research, the likelihood of any delays in recruiting patients to the study which might impact on the commencement date, and the likelihood of any delays in completing the contracting process.

   NOTE: In exceptional circumstances, we may be able to accommodate an earlier start date; however, this will be subject to approval from the Finance Team, and approval is not guaranteed.

2. **Trust contacts**
   Please provide the name, **email address** and postal address of:
   a. **the signatory at the Trust who will electronically sign the contract**
   b. the best person to copy into correspondence with regard to finance and contracting matters

3. **Collaborators**
   Please provide a list of the collaborators in the research and briefly outline the role each collaborator will have.

4. **Collaboration agreements**
   Please see condition 3.4 of the standard contract. We are aware that some organisations have long-standing collaboration agreements with certain partners/collaborators. If this is the case, please send me copies of these agreements for review.

   If there are no collaboration agreements currently in place, but agreements will be put in place in the future, please provide details of the collaborations to be established. Please include information on the number of collaboration agreements to be produced, who will be party to which, and estimated dates by which each agreement will be drafted. Once these agreements have been drafted, please send me copies for review.

5. **Subcontractors**
   If the research will involve sub-contractors, please provide the names of these and briefly outline the role each will play in the research.

IP Queries

1. **Background IP**
   Typically, research will use data, technology, materials and other inventions that may be (i) owned by a third party; and/or (ii) subject to rights of a third party (Background IP). Therefore, please consider:
   a. whether any permissions/licenses are required to use the Background IP for the Research; and
   b. the implications of any restrictions imposed by the owners and/or funders of the Background IP that may impact the Research and/or the potential future roll-out within the NHS and/or social care community.

   With this in mind, we draw your attention to the warranty at 23.1.5(a) of the research contract which states that the Contractor has an unrestricted and free right to use the Background IP for the purposes of the Research unless otherwise declared in Schedule B. Therefore, if there are any restrictions or encumbrances on the Background IP, please complete and return Schedule B below for NIHR's review. Alternatively, please confirm that you are able to agree to the warranty without having to disclose any encumbrances associated with the Background IP, in which case Schedule B can remain blank.

Description of Background IP	Owner of relevant Background IP	Nature of restriction	Risk to research and outcomes

2. **Foreground IP and Research Data**
   During the course of RfPB funded research, we would expect that some outputs will be generated, such as data, results, new interventions, scales or outcome measures. This Foreground IP and Research Data could be used directly in the NHS and/or via a third party through commercialisation so we require that you provide clear and appropriate Foreground IP and Research Data ownership arrangements. To this end we strongly recommend that you obtain advice from your contracts office and/or IP/technology transfer advisor as appropriate.

   Please list the Foreground IP and Research Data this research will generate or improve, such as the nature based intervention. As described in clause 15.2, **our starting position is that the Foreground IP and Research Data shall vest in the Contractor**, (Leeds and York Partnership NHS Foundation Trust). However, if you envisage having different arrangements for IP ownership, please provide a justification for why the proposed arrangement would be better suited to realise patient benefit. Either way, **please provide us with text to describe the Foreground IP and Research Data ownership arrangement(s)** because this will need to be set out in **Schedule C** of the contract (subject to any negotiations with our IP team).

3. **Project-specific Queries**
    - Please outline what involvement Yorkshire Wildlife Trust will have in this project.

4. **Commercialisation**
   As your application suggests possible commercialisation of the Foreground IP and/or data arising from this research, please provide confirmation that the Contractor and/or Collaborator has read and understood clause 16.5 of the standard contract which states that it/they will need to seek prior written consent from NIHR to do this.

I strongly recommend that you work with the Contractor's R&D and/or Technology Transfer Office to assist you with the above. If an appropriate draft of schedules B and C is not received by **02 December 2021** the contract will be prepared using our standard wording, with all foreground IP assigned to the Contractor.

Finance Queries

Alongside the scientific negotiations and intellectual property discussions currently underway, you may be also required to provide some further information regarding the application budget, which will be used to prepare the contract for this award. Please note that you will be contacted in regards to this separately by the CCF Finance Department (finance&contracts@nihr-ccf.org.uk).

I look forward to receiving your responses to the queries above.

Please do not hesitate to contact me should you have any queries or concerns.

With kind regards,

Stuart Haylock
stuart.haylock@nihr.ac.uk
02036927940

Research for Patient Benefit Programme

Welcome pack and overview of the grant holders' project monitoring responsibilities

Congratulations on your successful application to the Research for Patient Benefit (RfPB) Programme. We look forward to working with you. Please find below an overview of the main responsibilities that are required of you as a RfPB grant holder.

A Programme Manager in the RfPB team based at the National Institute for Health Research (NIHR) Central Commissioning Facility (CCF) will be assigned as a dedicated point of contact for your project and should be your first point of call for all project-related queries.

The contractual responsibilities of grant holders are described in the standard Department of Health and Social Care (DHSC) research contract. It is recommended that you familiarise yourself with all the terms and conditions of the contract and ensure that these are acted upon during the course of the research. This document contains the main actions that the Chief Investigator (CI) and/or contracting organisation will need to fulfil in order to satisfy the NIHR's project monitoring requirements.

1. Submission of favourable ethical opinion/Health Research Authority (HRA) approval

NIHR requires, where appropriate, evidence that an approval has been granted by the HRA. Please email copies of any relevant documentation to the CCF programme manager quoting your project's NIHR reference number. Favourable ethical approval does not need to be provided before the project starts, but will need to be in place before any patient recruitment commences.

2. Registration of studies

All NIHR-funded primary research studies are required to register in an appropriate registry. The NIHR's registry of choice is the International Standard Randomised Controlled Trial Number Register (ISRCTN). In some cases, other recognised registries may be appropriate or required for the primary registry, e.g. where there is a regulatory requirement. In such cases, NIHR will only require registration once, and will therefore waive the requirement for registration in ISRCTN. You can find further information at the ISRCTN website at www.isrctn.org. Please note that the remit of this database has been widened to include all primary research projects, even those that are not randomised controlled trials. You are required to register your trial prior to initiation of patient recruitment. NIHR Studies meeting certain criteria are eligible for free registration. Please provide your CCF programme manager with the ISRCTN number for this project once registered.

Registries must be updated during the study and key outcomes and trial protocols are to be made publicly available within 12 months from study completion. Trial findings should be published in a peer-reviewed journal or platform within 24 months from study completion. For further information see the NIHR policy on clinical trial registration and disclosure of results.

3. Project start

In order to ensure that payments for research projects are not made in advance of need,

RfPB has a formal process for start date confirmation. It is the CI's and the Trust's Research and Development (R&D) officer's responsibility to keep the CCF programme manager informed of any delays in starting the project on the contracted start date. To confirm the actual start date, the CCF programme manager requires an email from the Trust's R&D office to be sent to manager@ccfrms.org.uk, on or shortly after the date that the research activities officially commence, stating:

'This is to confirm that the NIHR funded award reference number started on insert actual start date'

DHSC wishes to see a dramatic and sustained improvement in the initiation of clinical research. In the event that the actual start date is more than three months after the contracted start date, the contracting organisation will be required to sign a variation to contract that will be issued by the CCF programme manager. Please be aware that to avert serious delays, a drop dead date (six months after the contracted start date) will be included within the contract to indicate the latest date by which the research must have started.

4. Completion of progress reports and financial statements

During the course of the project, grant holders are required to provide verbal or written reports of the technical progress on the project and details of the actual expenditure on the research activities. Template progress report forms will be made available either by email or through CCF's online research management system (RMS) https://ccfrms.nihr.ac.uk/Login. The report should convey an overview of progress made since any earlier report. The report is designed to capture any material changes to the research or staffing, a summary of progress to date, progress against the project milestones, a list of presentations and publications, background and foreground intellectual property (IP) positions and matters requiring further attention by the CCF programme manager.

Typically, the first progress report is expected 12 months after the actual start date for your project and subsequent reports are required at 12 month intervals. Where a project is contracted to run for 12 months or less, a progress report is required after six months. It is worth factoring the progress report due dates into your research plan.

Template annual statements of expenditure (ASTOX) will be issued following the start of each new financial year. These will need to be completed by the finance officer at the contracting organisation and should capture all the expenditure incurred by the contracting and partner organisations to deliver the research during the course of the preceding year. Please note that the contracting organisation is required to maintain proper financial records relating to the research at all times during the research period and for six years after the project's end. DHSC reserves the right to request further financial information about projects funded through the NIHR programmes at any time.

5. Informing CCF of anticipated changes (Variations to contract)

If at any time it appears likely that any amendments will be required to the contracted terms please contact your CCF programme manager about the nature and rationale for the anticipated change. The CCF programme manager may ask you to complete a variation to contract request form so that the proposed change can be considered by one of the programme's authorities. The following changes would require CCF approval and may also require a variation to contract:

1. Change of commencement date
2. Change of completion date (time extension)

3. Change of contracted amount (funding extension)
4. Change to research project (revision/addition/deletion of work)
5. Change of CI and/or co-applicants

If the change is approved, the CCF programme manager will issue a formal variation to contract to capture the change. This document will need to be signed by an authorised signatory at the contracting organisation and countersigned by DHSC. Please note that any request must be fully justified and approval is not guaranteed.

6. Publications and Outputs

To maximise awareness of the outputs of the research funded by the NIHR, nationally and internationally, grant holders are required to ensure that NIHR is named and acknowledged appropriately when submitting a paper or report for publication. Use of the correct project reference greatly aids the automated identification of publications and contributes to NIHR's ability to report accurately on the outputs, outcomes and impact of the work we fund.

Please ensure that the following statement is included in any presentations, posters or papers.

STARTS
This project is funded by the National Institute for Health Research (NIHR) under its Research for Patient Benefit (RfPB) Programme (Grant Reference Number NIHRXXXXXX). The views expressed are those of the author(s) and not necessarily those of the NIHR or the Department of Health and Social Care.
ENDS

For more information about how to acknowledge NIHR in your dissemination outputs, including how to find and use the 'Funded by NIHR' logo on your poster and conference presentations, please see **Appendix A** below. Grant holders, through their host institution communications teams, are **required to send details of all media activity** (e.g. press releases, media exclusive journalist briefings etc) to the relevant NIHR coordinating centre team. For further details please refer to **Appendix A** below.

NIHR supports the principle of publishing research findings as early as possible, but acknowledges that publication in a peer-reviewed journal or platform is not within the complete control of the research team and has therefore set an indicative timeframe of publishing findings within 24 months from study completion.

NIHR uses Researchfish to monitor the outputs of funded research, which is published in peer-reviewed journals. On an annual basis, NIHR-funded researchers are asked to submit data about their outputs, outcomes and impacts. The data submitted allows NIHR to demonstrate the difference that a funded research project has made to the health and wealth of our nation; please visit NIHR impact page for further information.

For further comms advice for new award holders, please refer to Appendix A below.

7. Intellectual Property (IP)

Grant holders are expected to be aware of the research contract's terms and conditions in relation to the IP rights. In particular, the grant holder is required to inform the CCF programme manager of any results which are capable of being exploited either by direct adoption into the healthcare service or via commercialisation. The contracting organisation

will also need to seek prior written approval from CCF if it wishes to use a third party (excluding professional advisors) to carry out exploitation activities with respect to the foreground IP funded through this grant. CCF is interested in following up on the impact of research funding, so further information on directly exploitable IP arising from your project may be requested once the project is complete.

8. Completion of a final report and final statement of expenditure (FSTOX)
A draft final report containing all of the analyses anticipated in the original application will need to be submitted within 14 calendar days of the contracted end date. A template final progress report form will be made available on our online research management system (RMS) 8 weeks in advance of the due date. Within the final progress report you will be expected to give a comprehensive overview of the work undertaken, including a plain English summary, summary of the research findings (including background, aims and objectives, methodology, key findings, expected impact and conclusions), outline any variations from the original proposal, next steps to patient benefit and the dissemination strategy. RfPB is particularly interested to learn about the experiences of research teams stemming from the patient and public involvement (PPI) in their projects, so further information on PPI in your project may be requested once the project is complete.

The final statement of expenditure (FSTOX) will be made available alongside the final report and this needs to be completed and signed by an authorised representative of the contracting organisation administering the grant. Both reports are a requirement and must be submitted before the final payment, usually 10% of the total award, can be released.

9. Post close project monitoring
During the process of closing the project, the CCF programme manager and CI should discuss the expected post close short-term and long-term outcomes, and the estimated timelines. Examples of possible project outcomes include: publication in peer reviewed journals, press releases, conference presentations, recommendations for NICE guidelines, further funding sought for feasibility and more upstream (Tier 3) projects, any patents/copyrights/trademarks filed and/or granted, formation of a spin-off company, clinical dissemination etc. Taking into account the expected timelines for short and long-term project outcomes, the CCF programme manager and CI will agree on the frequency and mode of contact for post award monitoring, e.g. call or email every 6 months in the first two years and then annually for the next 3-5 years.

Appendix A.

Comms advice for new award holders

Congratulations on your successful application for funding from the NIHR. This document provides a summary of key NIHR guidance to bear in mind at the start of your project. It covers NIHR branding and acknowledgement requirements, as well as advice on digital channels and the requirements for media relations for your NIHR-funded research.

The NIHR also has requirements around reporting research outputs as a project progresses, such as:

- Publications, including journal articles
- Engagement activity based outputs, such as talk or presentations
- Policy, practice, patients and the public outputs, such as policy documents and participant materials
- Research databases and models
- Intellectual property

Please review the NIHR research outputs and publications guidance for full details.

NIHR brand requirements

We encourage you to use the 'Funded by NIHR' logo on visual and digital outputs, along with acknowledging the NIHR and using our disclaimer.

Research teams are permitted to create logos for their NIHR-funded studies, providing the NIHR acknowledgement is also in place.

'Funded by NIHR' logo

FUNDED BY
NIHR | National Institute for Health Research

The 'Funded by NIHR' logo should be used along with our acknowledgement and disclaimer:

- On websites, above the fold but not in the header, i.e. so that it is visible without scrolling down the page
- In print, such as on research posters and patient leaflets, below the fold/in the bottom half of the page
- In research slides, with the ‘Funded by NIHR’ logo on the first slide and the acknowledgement and disclaimer on the final slide.

Download a copy of the 'Funded by NIHR' logo.

Funded researchers should use their institution's templates for research posters and slide decks. Alternatively, for research posters, you can use an NIHR poster template and add your host institution logo.

The 'Funded by NIHR' logo should not be used on:
- Original research papers
- Press releases

Funded researchers should not use the corporate NIHR logo.

More information is available on the outputs and branding page on the NIHR website.

Acknowledging the NIHR

All written and oral research outputs should acknowledge the NIHR funding in full, include the NIHR unique award identifier and include the NIHR disclaimer.

Please use the following wording to acknowledge the NIHR:
This study/project is funded by the National Institute for Health Research (NIHR) [name of NIHR programme (NIHR unique award identifier)]. The views expressed are those of the author(s) and not necessarily those of the NIHR or the Department of Health and Social Care.

Please also acknowledge any other NIHR support your project may have received; for example, from our Clinical Research Network or an NIHR Clinical Research Facility or Biomedical Research Centre.

Read more about acknowledging the NIHR in research outputs in the NIHR research outputs and publications guidance.

Digital channels for your project

Award holders are permitted to create digital channels for their NIHR-funded research project, such as websites and social media accounts.

Websites

Websites should display the 'Funded by NIHR' logo should be used above the fold but not in the header, i.e. so that it is visible without scrolling down the page.

Websites should also use the NIHR acknowledgement and disclaimer.

Social media accounts

Award holders are permitted to create social media accounts for their research project, such as Twitter, YouTube and Facebook.

Social media accounts should acknowledge the NIHR funding in their bio. For example, tagging the NIHR Research twitter account (@NIHRresearch) in a twitter bio:

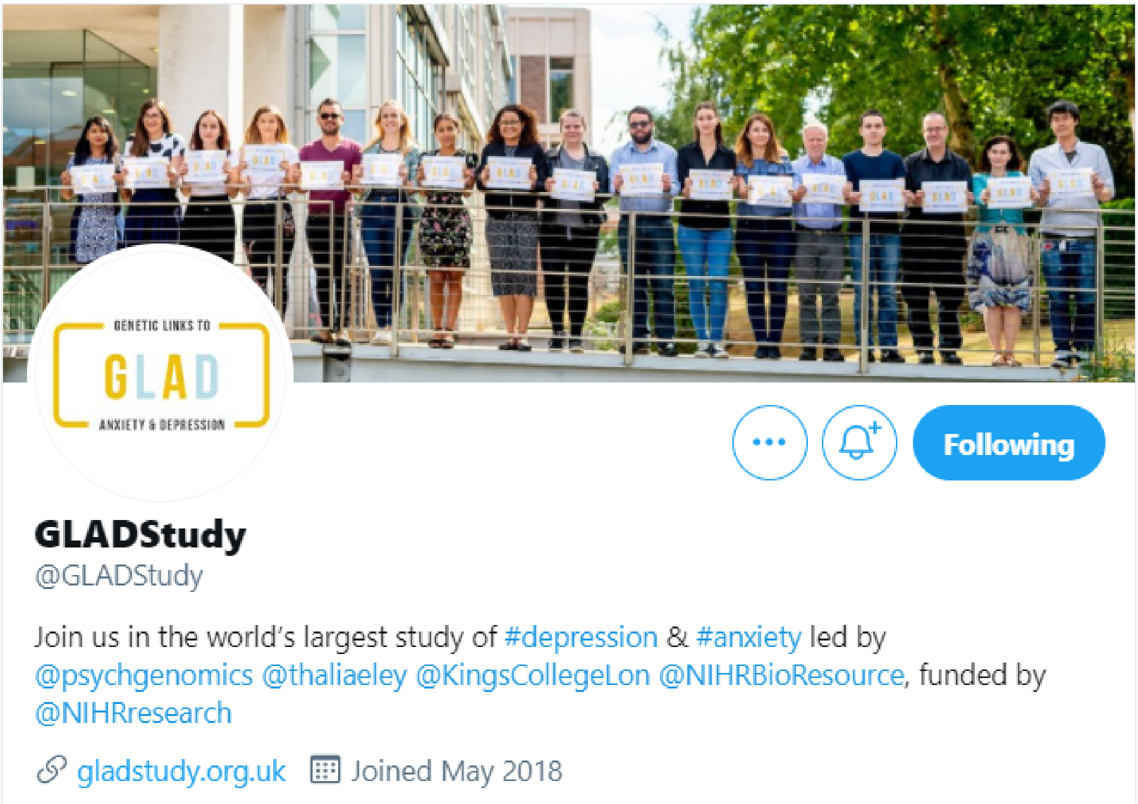

We also recommend that you tag your host organisation in any social media bios.

The NIHR has a number of audience-focused social media streams that you can follow, supplemented by topic specific streams. You can get the latest news and updates from the NIHR delivered to your inbox by signing up to our monthly newsletters.

We would like to encourage you to engage with and re-share NIHR content on your own channels to help amplify the work of the NIHR, as well as creating unique posts publicising your funded research. Tag us in your posts about your research and we may be able to re-share and amplify too.

Media relations

Award holders are required to notify **all media activity** to the communications team at the NIHR Central Commissioning Facility (CCF) via ccfcomms@nihr.ac.uk. This notification may be by the award holder themself or through their host institution communications team.

Media activity that should be notified to the NIHR includes:

- A press release being issued through the researchers' host or partner institution
- A press release being issued by the journal, funder or partner in the research
- A researcher taking part in interviews for press or broadcast

- A media exclusive or journalist briefings

This notification should take place as soon as reasonably practicable and a **minimum of three working days prior to any journalist outreach**. Notification should include copies of all press releases and, where possible, the relevant research papers.

Press releases should acknowledge the NIHR funding and include the NIHR boilerplate in the notes to editors. Full details are available in the NIHR research outputs and publications guidance.

Questions and help

The CCF communications team are available for advice on ccfcomms@nihr.ac.uk.
