# Supplementary material for: Co-production of a nature-based intervention for children with ADHD study (CONIFAS): Protocol for co-production phases
Source: PLoS One. 2022 Sep 20;17(9):e0274375. doi: 10.1371/journal.pone.0274375 (PMC9488762; doi:10.1371/journal.pone.0274375)
Supplement: S4 File — The reviews conducted by anonymous peer reviewers as part of the NIHR RfPB funding scheme. (PDF) [file pone.0274375.s004.pdf]

NIHR | National Institute for Health Research

Research for Patient Benefit Review Summary

Reference Number	NIHR203043
Research Title	Development of a nature based intervention for children with ADHD (NICA)
Chief Investigator	Dr Hannah Armitt
Contracting Organisation	Leeds and York Partnership NHS Foundation Trust
Total Amount Requested	£149,599.00
Reviewer Number	1

Table Of Contents

1. Reviewer Expertise
2. Relevance of the proposed research
3. Quality of the proposed work
4. Strength of the research team
5. Impact of the proposed work
6. Value for money
7. Involvement of patients and the public
8. Additional Comments

1. Reviewer Expertise

**Please indicate the nature of your expertise by clicking on the appropriate tick box(es) below:**
Clinician in a broadly related field, Researcher in a broadly related field

If the tick boxes above do not adequately capture the nature of your expertise, please briefly provide details in the box below (or use it to give us more detail about your expertise if you wish):

2. Relevance of the proposed research

i) How relevant and important is the proposed research to the priorities and needs of the NHS, and does it offer a health/healthcare solution with demonstrable benefit to patients?<br>ii) Does the application demonstrate an awareness and understanding of previous relevant research or developments in this area?<br>iii) To what extent does the proposed research add distinct value or advance existing knowledge in this area, taking into account wider ongoing or completed research?
i) this proposal does address a clear clinical need, which given limited access and appropriateness of existing interventions, the focus on children and families, plus the higher prevalence in low income households is a priority. If the intervention is feasible and shown to work there will be benefit to patients.<br><br>ii) The prevalence, clinical need and service context of children with ADHD and their families in the UK was well described. However, there is more in the research literature which could have been drawn on from existing studies to evidence what has already been found about the use of green spaces to reduce ADHS symptomology e.g. Taylor et al, (2014) doi:10.1111/j.1758-0854.2011.01052.x; Barfield et al (2018) doi.org/10.1111/jspn.12210. This would have been useful to provide a foundation of what has already been found effective in interventions with similar intentions.<br><br>iii) This is a novel area which could have patient benefits if successful, and is efficient in making use of exiting community assets. I think it also offers the nuance required to make some of the more generic policies to 'get active' and use nature more, beneficial to groups which have specific clinical needs. In the UK I am not aware of any similar services or projects tailored to this group.

3. Quality of the proposed work

**Research Design**<br>i) How appropriate is the research design in relation to the stated objectives?<br>ii) To what extent is the proposed design and methodology for all elements of the research well defined, appropriate, valid and feasible within the timeframe and resources requested?<br>iii) What are the strengths and weaknesses of the research design as proposed?<br>iv) To what extent does the research show originality and innovation?
i) the general design is appropriate but there is some 'drift' in how the objectives and research questions are articulated. In section three the research question is posed as' what are the core components of a nature based intervention' for this group. Later the aims suggests the purpose is to 'manage the impact of ADHD symptoms on their lives', and later (section 4) to 'reduce symptoms whilst developing skills to control the impact of ADHD'. This is important as it has consequences for the design. For example, in the design there is no mention of data being collected on change in management/skill level, whilst this does appear as an aim in section 4.<br><br>ii) the project is feasible to be carried out in the timeline within the described resource allocation. It should be possible to recruit participants as described. If the focus is clarified as mentioned above it should deliver the objectives.<br><br>iii) The aims needs to be more focussed and relate more clearly to the data to be collected. Existing past research needs to be used more effectively to feed ideas into the co-production of the intervention. The co-production and engagement of relevant stakeholders is a strength.<br><br>iv) There is some previous research on green spaces as an intervention for ADHD, although not so much in the UK. One of the strengths of this proposal is the embedded nature of the project within existing organisations and policies, bringing together those from health, social and environmental sectors. However, I was a little disappointed at the proposed outputs which seemed to be the intervention described in a leaflet and some written guidance. I would have hoped to see more enhanced outputs which sustain and build on these allegiances.

Has the research been designed with reference to an appropriate review of the existing literature?
Yes

**Work plan and proposed management arrangements**<br>i) How appropriate are the work plan and project management arrangements, and do they give confidence that proposed milestones will be met within the specified timeframe?<br>ii) Are the necessary clinical, academic or organisational links needed to support the research, in place?<br>iii) Please assess whether the key risks identified by the applicants have been adequately addressed, such as:<br><br>• Ethical, scientific, technical and organisational challenges<br>• Intellectual Property (IP) and commercial issues<br>iv) Please identify any additional barriers to the proposed work, not mentioned in the application form, that the applicants are likely to encounter in meeting their milestones<br>v) If this application has been submitted to the NIHR Programme Grants for Applied Research Programme, what added value would be delivered over and above the dividends from the individual elements?
i) A substantial team of appropriate stakeholders have been brought together to support the project. The design is not complicated and the participants numbers whilst small are appropriate and achievable. Given the earlier comment to the researchers in relation to round 1 of the need to take account other siblings in the family I was unclear if non-ADHD siblings would be included in the different phases, as it was stated dyads of the child with ADHD and a 'family member' would be included. Given that attention was paid in the rationale to the need to consider a range of family formats, and the role siblings play in these dynamics I would have thought they should be included somewhere within the design. Families will need to support the child with ADHD in delivering the

intervention, which means in reality it will also be delivered to or supported by siblings, and so the acceptability to them also needs to be judged.<br><br>ii) this is a strength of the project.<br><br>iii) I think the main risk of the impact of Covid as been identified, but I was not entirely convinced by the mitigation which was that previous trials have overcome this in the past (?) and that meetings could be recorded or go on line. No consideration seems to have been given to the impact on families should for example a national lock down be re-instituted, or that the material circumstances of the family substantially changes.<br><br>iv) Non that I can think of.<br><br>v) N/A

**Plain English summary**<br>The plain English summary is intended for an interested audience, who are not necessarily specialists. The summary should be written at roughly the same level as an article in a newspaper. With this in mind, please comment on the following:<br><br>i) Does the plain English summary give a clear explanation of the research?<br>• Does it help you carry out your review? If not, why not?<br>• Is the language used appropriate and clear? If not, where are the problems?<br>• Are scientific terms, abbreviations and jargon explained? If not, which terms need explanation?<br>ii) If this research is funded, the plain English summary will be published on a variety of websites, without the rest of this application form. Could this plain English summary be used on its own to describe the proposed research? If not, what further information is needed?<br><br>Further information for researchers on how to write a plain English summary and what to include in a summary is available online at NIHR Make it clear http://www.invo.org.uk/makeitclear/.
i) again the aims of the project are articulated slightly differently - this time being described as a 'before and after' study, introducing a new variable of 'mental health' symptoms. This shifting focus makes it hard to judge if the design will really deliver the aims. In different places in the proposal it describes a 'feasibility' study following this one. It would have been helpful to describe this study in the context of this progression from this proposal to a feasibility study to then a effectiveness study, or a RCT ? What framework is being used here ? Is this a 'proof of concept study'?<br><br>Some abbreviations need to be given in full e.g. COMIC/LYPFT<br><br>ii) not until there is consistency in the research objectives and fit the proposed design. The language used and general structure is understandable, and nice to see the positive aspects of ADHD also being mentioned. The term 'green treatment options' probably needs explanation.

4. Strength of the research team

i) How well are the roles of the team members described? Is the overall team well coordinated?<br>ii) On the basis of track record in relevant areas, how qualified are the applicants to undertake the work using the methodologies proposed?<br>iii) To what extent does the research team have the necessary breadth and depth of expertise to deliver the proposed work (*e.g.* as judged by publication output and previous research funding)?<br>iv) How could the strength of the research team be improved?<br>v) If the lead applicant is inexperienced, does he/she have appropriate support (*e.g.* from their organisation and/or more senior colleagues) to deliver the work plan?
i) roles are clearly described and the applicants are appropriately qualified.

ii) this should not be a problem, especially under the guidance of Prof Wright.

iii) Cannot judge as no publication lists or previous funding included in section 12.

iv) There is a good range of sectors represented, but I wonder if this could be added to by bringing in somebody with experience of delivery and evaluation of an aligned 'green project' e.g. a gardening project for mental health service users. This may add some practical, experiential advice in terms of delivering this project.

v) yes.

5. Impact of the proposed work

Dissemination, outputs and anticipated impact
NIHR aims to fund research that has the potential to be of significant benefit to the NHS, patients and the public. To support this, the applicants should consider how they will achieve impact from the outset. This helps them to identify potential impact and beneficiaries, and plan processes by which the research can directly, or incrementally over time, lead to change.

i) Have the applicants clearly expressed the problem and outlined how this research contributes towards a solution? Are the planned outputs appropriate and sufficient?
ii) Is it clear from the application who or which groups (including, as appropriate, healthcare planners, clinicians, patients and/or policy makers) are expected to benefit the most, how they will be engaged and communicated with, and if appropriate methods of engagement and dissemination are planned?
iii) Have the applicants set out appropriate activities and resources to achieve their impact goals? Is there a realistic trajectory and estimate of timescales for the benefit to reach patients/ public/ health and care services? Are there clear connections between outputs, engagement processes and impact goals?
iv) Have the applicants clearly considered what follow-on support they might need to generate or upscale impact, and how they might leverage further investment?
v) Have the applicants sufficiently and correctly identified any potential barriers they might face (e.g. IP, regulatory and acceptability) and have they properly considered how these may be overcome?
vi) Have the applicants clearly and realistically outlined the anticipated impacts, the likely scale of these impacts (both in the shorter and longer terms), and the sequence in which the impacts might occur?

i) They have adequately delineated why a project such as this is needed, but they have not really described the specific advantages of this type of intervention in for example, the mode of delivery or use of existing community assets. I find it hard to envisage how the outputs (a leaflet and guidance) will lead to a successful or sustainable intervention, which will be actively embraced by those who will deliver it. Indeed, I am unsure of what the infra-structure would be to sustain this intervention - who will actually deliver it? who will support this delivery? How will the stakeholders be brought together and sustained ? The systemic context of the intervention is not described adequately. I do not expect this project to answer all these questions but I expected more consideration to have been given to these issues. In my view the proposed outputs as described are insufficient given the investment in the project. Similar projects have produced a tool-kit or compendium of resources tailored to the needs of the stakeholders.

ii) The general design, engaged stakeholders and timescales will deliver the described project, but I think more resources in terms of outputs could be developed which would result in future sustainability.

iii) No, for me this is a major gap. A feasibility study is mentioned but it is unclear what this means, and what needs to be in place to move the work from this project to that.

v) Some have been identified but as mentioned previously I do not think the Covid context has been considered sufficiently and mitigating procedures described adequately.

vi) Again there seems to be a lack of longitudinal planning in terms of leveraging consequences resulting from any impact the project may happen. A Select Committee is being targeted to present the project - but what are the aspirations from this? It would be good to see this project more embedded in a theme of work endorsed by the

main stakeholders,

6. Value for money

i) Taking into consideration the costs associated with undertaking the research, is there sufficient justification for the resources requested? Are all costs essential for the work proposed?<br>ii) Are appropriate resources set aside for patient and public involvement - including plans for a training and support budget?<br>iii) Where relevant, are resources to support impact - other than those for public and patient involvement - included and appropriate?<br>iv) Taking into account the total cost of the research, including the NHS costs, to what extent does the research provide value for money?<br>v) If required, are funds requested for NHS support and treatment costs appropriate and justified?
i) I think the costs suggested are reasonable. My only concern is that the commitment of 15% of time by Armitt might be low in terms of the expertise and direction needed to deliver the project. Whilst the bulk of the delivery of the project will be delivered by the 1.0 RA and 0.5 trial co-ordinator, I wonder if the lead applicant will have sufficient time to manage this and attend to the stakeholder maintenance and development required.<br><br>ii) Yes, I think this is a strength.<br><br>iii) I think this could be enhanced, perhaps through a designated launch, or a 'see and try' event, or perhaps 'piggy backing' on an existing event to promote the outcomes.<br><br>iv) Currently, whilst I think the project is innovative and worth while I feel that confused focus and lack of embeddedness in a project development pathway detracts from the value for money. If these can be enhanced I think it would deliver good value for money.<br><br>v) N/A

7. Involvement of patients and the public

**Was there any patient and public involvement in the application?**	Yes

i) What is your assessment of the patient and public involvement (if any) in the development of the application, including involvement in: Identifying the research topic; prioritising the research questions; preparing the application (e.g. contributing to the research design); and identifying potential impact?<br>ii) What is your assessment of any proposed plans for patient and public involvement throughout the life of the research? Can you identify particular strengths, weaknesses and/or areas for improvement?
i) I think this is a strength of the project, and especially good to see the work gone into developing this proposal.<br><br>ii) I think this is generally good. My only reservations are around the three previously raised topics<br><br>- mitigations, especially for families re Covid<br><br>- lack of structural embeddedness leading to concerns about sustainability<br><br>- limited outputs, not really tailored to support the network required to deliver and sustain the intervention.

8. Additional Comments

If you have any additional comments, not covered by the sections above, please provide them below.
No further comment

NIHR | National Institute for Health Research

Research for Patient Benefit
Review Summary

Reference Number	NIHR203043
Research Title	Development of a nature based intervention for children with ADHD (NICA)
Chief Investigator	Dr Hannah Armitt
Contracting Organisation	Leeds and York Partnership NHS Foundation Trust
Total Amount Requested	£149,599.00
Reviewer Number	2

Table Of Contents

1. Reviewer Expertise
2. Relevance of the proposed research
3. Quality of the proposed work
4. Strength of the research team
5. Impact of the proposed work
6. Value for money
7. Involvement of patients and the public
8. Additional Comments

1. Reviewer Expertise

Please indicate the nature of your expertise by clicking on the appropriate tick box(es) below:
Researcher in a broadly related field

If the tick boxes above do not adequately capture the nature of your expertise, please briefly provide details in the box below (or use it to give us more detail about your expertise if you wish):

2. Relevance of the proposed research

i) How relevant and important is the proposed research to the priorities and needs of the NHS, and does it offer a health/healthcare solution with demonstrable benefit to patients?<br>ii) Does the application demonstrate an awareness and understanding of previous relevant research or developments in this area?<br>iii) To what extent does the proposed research add distinct value or advance existing knowledge in this area, taking into account wider ongoing or completed research?
- As stressed in the proposal, this research is relevant as it offers additional intervention options for children with ADHD and their parents. There are enormous waiting lists so this would offer potential in that context. However, the following elements could be strengthened in the proposal:<br><br>- What are the mechanisms on how green spaces contribute to mental health? It feels like more fundamental research needs to be described.<br><br>- How would this intervention be specifically effective for children with ADHD? As I would think it would benefit any child mental health (in such a busy, performance focused, digital era) so why not broaden it for other (neurological) disorders?<br><br>- What is currently available for children with ADHD alongside medication? Is exploring nature not already part of regular school curriculum or even forest schools at nurseries?<br><br>- It is stated that the aim of the intervention is to reduce symptoms while developing skills to control impact of

ADHD on their lives – so it is expected that these skills will mediate effects the intervention has on ADHD symptoms?

3. Quality of the proposed work

Research Design
i) How appropriate is the research design in relation to the stated objectives?
ii) To what extent is the proposed design and methodology for all elements of the research well defined, appropriate, valid and feasible within the timeframe and resources requested?
iii) What are the strengths and weaknesses of the research design as proposed?
iv) To what extent does the research show originality and innovation?

- Please clarify terminology of coproduction and co-design (mentioned on page 16)

- It would be good to see how the Intervention Mapping Framework would be useful in the context of intervention development

- The research seems feasible in the timeframe and resources requested (with COVID being so unpredictable there might be a case for extending with 6 months)

- The co-production methodology follows adequate frameworks and use of tools. It would be interesting to get a bit more detail on how data from the discovery groups and workshops will be analyzed, also regarding the diaries that have been added

- I am just wondering if it should be attempted to have a workshop with children (in which parents are present but do not deliver input as such) so they can provide their own input without feeling overruled by their parents

- It might be good to consider adding the teacher CONNORS version too as ADHD normally displays itself in home and school context (as part of the diagnosis)

- It would be good to explicitly indicate if the satisfaction survey is self-constructed or not

- It would be good if main difficulties for children with ADHD could be identified in the literature so this can be used to discuss more in-depth during the discovery groups.

- Co-production is almost like a basic requirement when developing interventions nowadays so the main innovation is in the intervention approach. However, I am not fully convinced this is specifically useful for children with ADHD and would like to see a better rationale and description of fundamental research describing how green spaces improve mental health

- Would a measure of self-efficacy be considered for children?

- Would a digital format of a daily diary method, such as EMA, be considered for data collection?

Has the research been designed with reference to an appropriate review of the existing literature?
Yes

Work plan and proposed management arrangements
i) How appropriate are the work plan and project management arrangements, and do they give confidence that proposed milestones will be met within the specified timeframe?
ii) Are the necessary clinical, academic or organisational links needed to support the research, in place?

iii) Please assess whether the key risks identified by the applicants have been adequately addressed, such as:<br><br>• Ethical, scientific, technical and organisational challenges<br>• Intellectual Property (IP) and commercial issues<br>iv) Please identify any additional barriers to the proposed work, not mentioned in the application form, that the applicants are likely to encounter in meeting their milestones<br>v) If this application has been submitted to the NIHR Programme Grants for Applied Research Programme, what added value would be delivered over and above the dividends from the individual elements?
- The proposal entails an appropriate work plan and project management plan which gives confidence that milestones (these should be made more clear in the proposal; milestones is only mentioned once) will be met within the timeframe (as mentioned previously an extension of 6 months might be needed depending on COVID)<br><br>- All expertise seems to be represented across the team and relevant organizations are lined up<br><br>- It is good that online means are considered to prevent delays due to COVID. However it is not clear if this only concerns the management meetings or includes co-production workshops (which would not be recommended to be performed online). How are pre- and post-test surveys collected? Will this be done online too?<br><br>- As NHS professionals will support recruitment it seems to me that IRAS ethical approval is needed which normally takes longer than 4 months. If only local ethical approval is needed than it would be good to indicate why and use the HRA tool to demonstrate it is about service evaluation for example<br><br>- It might be good to stress different consent forms for different participant for example written informed consent from adults and assent from children

**Plain English summary**<br>The plain English summary is intended for an interested audience, who are not necessarily specialists. The summary should be written at roughly the same level as an article in a newspaper. With this in mind, please comment on the following:<br><br>i) Does the plain English summary give a clear explanation of the research?<br>• Does it help you carry out your review? If not, why not?<br>• Is the language used appropriate and clear? If not, where are the problems?<br>• Are scientific terms, abbreviations and jargon explained? If not, which terms need explanation?<br>ii) If this research is funded, the plain English summary will be published on a variety of websites, without the rest of this application form. Could this plain English summary be used on its own to describe the proposed research? If not, what further information is needed?<br><br>Further information for researchers on how to write a plain English summary and what to include in a summary is available online at NIHR Make it clear http://www.invo.org.uk/makeitclear/.
The plan English summary is clear and sufficient. Ideally you want to know which age group of children with ADHD the intervention is focusing on.

4. Strength of the research team

i) How well are the roles of the team members described? Is the overall team well coordinated?<br>ii) On the basis of track record in relevant areas, how qualified are the applicants to undertake the work using the methodologies proposed?<br>iii) To what extent does the research team have the necessary breadth and depth of expertise to deliver the proposed work (*e.g.* as judged by publication output and previous research funding)?<br>iv) How could the strength of the research team be improved?<br>v) If the lead applicant is inexperienced, does he/she have appropriate support (*e.g.* from their organisation and/or

more senior colleagues) to deliver the work plan?
- It seems that the research assistant (and this person is not identified yet?) does most of the field work and is supervised by more senior team members. I am just wondering to what degree the PI has experience with co-production herself as this seems to be quite crucial for a successful outcome and how a successful delivery can be guaranteed by the RA<br><br>- Prof Wright seems to have the right expertise to supervise the lead applicant

5. Impact of the proposed work

**Dissemination, outputs and anticipated impact**<br>NIHR aims to fund research that has the potential to be of significant benefit to the NHS, patients and the public. To support this, the applicants should consider how they will achieve impact from the outset. This helps them to identify potential impact and beneficiaries, and plan processes by which the research can directly, or incrementally over time, lead to change.<br><br>i) Have the applicants clearly expressed the problem and outlined how this research contributes towards a solution? Are the planned outputs appropriate and sufficient?<br>ii) Is it clear from the application who or which groups (including, as appropriate, healthcare planners, clinicians, patients and/or policy makers) are expected to benefit the most, how they will be engaged and communicated with, and if appropriate methods of engagement and dissemination are planned?<br>iii) Have the applicants set out appropriate activities and resources to achieve their impact goals? Is there a realistic trajectory and estimate of timescales for the benefit to reach patients/ public/ health and care services? Are there clear connections between outputs, engagement processes and impact goals?<br>iv) Have the applicants clearly considered what follow-on support they might need to generate or upscale impact, and how they might leverage further investment?<br>v) Have the applicants sufficiently and correctly identified any potential barriers they might face (e.g. IP, regulatory and acceptability) and have they properly considered how these may be overcome?<br>vi) Have the applicants clearly and realistically outlined the anticipated impacts, the likely scale of these impacts (both in the shorter and longer terms), and the sequence in which the impacts might occur?
The pathway to impact has been described clearly. Follow-up funding opportunities for the RCT trial could be added. It might be worthwhile to consider offering elements of the intervention to children on the waiting list (as 6 years seems to be quite a long stretch to wait for its full-blown evidence-based status) and could potentially benefit some people.

6. Value for money

i) Taking into consideration the costs associated with undertaking the research, is there sufficient justification for the resources requested? Are all costs essential for the work proposed?<br>ii) Are appropriate resources set aside for patient and public involvement - including plans for a training and support budget?<br>iii) Where relevant, are resources to support impact - other than those for public and patient involvement - included and appropriate?<br>iv) Taking into account the total cost of the research, including the NHS costs, to what extent does the research provide value for money?<br>v) If required, are funds requested for NHS support and treatment costs appropriate and justified?
All costs seem essential for the work proposed and the proposed research provides value for money although the intervention scope has potential to broaden its audience.

7. Involvement of patients and the public

**Was there any patient and public involvement in the application?**	Yes

i) What is your assessment of the patient and public involvement (if any) in the development of the application, including involvement in: Identifying the research topic; prioritising the research questions; preparing the application (e.g. contributing to the research design); and identifying potential impact?<br>ii) What is your assessment of any proposed plans for patient and public involvement throughout the life of the research? Can you identify particular strengths, weaknesses and/or areas for improvement?
Patient and public involvement seems adequate and ensured throughout the research cycle of the current project. It is very powerful that a PPI member is a member of the research team. I would add discussing the frequency of activities in the intervention with the PPI members as 5 per week seems a like a lot.

8. Additional Comments

If you have any additional comments, not covered by the sections above, please provide them below.
- Is there a need for translation support for BAME community members involved?

NIHR | National Institute for Health Research

Research for Patient Benefit Review Summary

Reference Number	NIHR203043
Research Title	Development of a nature based intervention for children with ADHD (NICA)
Chief Investigator	Dr Hannah Armitt
Contracting Organisation	Leeds and York Partnership NHS Foundation Trust
Total Amount Requested	£149,599.00
Reviewer Number	3

Table Of Contents

1. Reviewer Expertise
2. Relevance of the proposed research
3. Quality of the proposed work
4. Strength of the research team
5. Impact of the proposed work
6. Value for money
7. Involvement of patients and the public
8. Additional Comments

1. Reviewer Expertise

**Please indicate the nature of your expertise by clicking on the appropriate tick box(es) below:**
Researcher in a broadly related field

If the tick boxes above do not adequately capture the nature of your expertise, please briefly provide details in the box below (or use it to give us more detail about your expertise if you wish):

2. Relevance of the proposed research

i) How relevant and important is the proposed research to the priorities and needs of the NHS, and does it offer a health/healthcare solution with demonstrable benefit to patients?<br>ii) Does the application demonstrate an awareness and understanding of previous relevant research or developments in this area?<br>iii) To what extent does the proposed research add distinct value or advance existing knowledge in this area, taking into account wider ongoing or completed research?
This is an important area of research. With ever increasing waiting lists and demand outstripping capacity there is a clear need to offer interventions outside a traditional CAMHS setting. There is sufficient background knowledge that indicates that green space and outdoor activities may be particularly beneficial for ADHD children which is outlined in the application and i think this is an interesting and worthwhile area to investigate.<br><br>The application extends existing knowledge by integrating this in CAMHS, which to the best of my knowledge has not been done. Although as part of the reviewers feedback from Stage 1, the applicants address how this may fit in one CAMHS service, i would like to see how this fits more generally in the CAMHS care pathway, or even if this service should be offered pre-CAMHS (school, GP?) or as a wait-list intervention? I would also like to see a recognition of what you feel this may replace or will it be an adjunct to standard care? Stimulant medication is still recommended 1st line treatment which isn't given enough consideration in the background.

My concern would be how generalisable the intervention may be to CAMHS based in large cities and urban areas. Particularly for families of lower SES and lack of access to transport. I see as part of the workshops professionals from Northern CAMHS will be recruited. Representation from urban and rural areas would be important. The applicants state they will recruit a diverse sample, including a range of SES this would be key to ensuring that the findings/outputs from this study are relevant and generalisable.

3. Quality of the proposed work

Research Design
i) How appropriate is the research design in relation to the stated objectives?
ii) To what extent is the proposed design and methodology for all elements of the research well defined, appropriate, valid and feasible within the timeframe and resources requested?
iii) What are the strengths and weaknesses of the research design as proposed?
iv) To what extent does the research show originality and innovation?

I am pleased to see that the methodology follows the Double Diamond Model, an established method and the research design is relevant to the stated objectives. There is a clear method explained how the phases will lead to the development of an intervention.

The method is relatively well described, although information about who will make the first approach to families and how they will be identified could be expanded, will this be a database trawl of people meeting the criteria? Will you record numbers approached vs numbers accepted as a measure of generalisability. I also was a bit confused about the ethics regarding participant recruitment. If you recruit from the NHS, i think REC not university ethics should be sought. I did not feel that sufficient time was given for study set up including ethics , staff recruitment and participant recruitment. Identifying and engaging with the CAMHS sites would also require time and there was not enough consideration given to this. I realise the numbers recruited are relatively small, but recruiting to these types of groups will still be challenging.

I would like to see some consideration given to alternative methods of meeting if COVID restrictions do not allow face-to-face meetings at the time of the study delivery. My previous experience of conducting work with ADHD families also indicates that CYP often will NOT attend face-to-face meetings. I think the researchers should consider "plan B" methods if this is problematic. Particularly involving CYP and professionals in the same meeting. Consideration should also be given as to how younger CYP may be involved. Involving a 5 year old in this feels challenging to say the least! On this note, you say you will gather consent from CYP and parents, but given the age of your CYP, their assent would be more appropriate.

The applicants outline their measures but there is no measure of Quality of Life (QoL), which i would consider a potential important outcome? In general, i would seek PPI feedback on the choice of measures (perhaps this has been done?). For example, why was Conners selected over SNAP-IV? Along with this it would be good to reflect on what you would feel would be the primary outcome in a later study, ie, what is the primary intent of the intervention? To improve ADHD symptoms? All or (for eg) hyperactivity more so? Or perhaps to improve something more generic like QoL. I think this also links to a more general issue that i would like it be more clear what the intervention would aim to improve.

A strength to this research is the good research team that have the skills and experience to deliver the study. Furthermore, the continuous involvement of PPI is to be commended. The study uses an established method in order to investigate their aim.

The research topic is novel and has potential to inform interventions offered by CAMHS (or other services). The proposed application would be a required and necessary step in order to further investigate this. Weaknesses are: time scale and ethical approvals required, lack of detail on the broader picture of where this sits in services/aim of intervention

Has the research been designed with reference to an appropriate review of the existing literature?
Yes

**Work plan and proposed management arrangements**<br>i) How appropriate are the work plan and project management arrangements, and do they give confidence that proposed milestones will be met within the specified timeframe?<br>ii) Are the necessary clinical, academic or organisational links needed to support the research, in place?<br>iii) Please assess whether the key risks identified by the applicants have been adequately addressed, such as:<br><br>• Ethical, scientific, technical and organisational challenges<br>• Intellectual Property (IP) and commercial issues<br>iv) Please identify any additional barriers to the proposed work, not mentioned in the application form, that the applicants are likely to encounter in meeting their milestones<br>v) If this application has been submitted to the NIHR Programme Grants for Applied Research Programme, what added value would be delivered over and above the dividends from the individual elements?
I am concerned about some of the timing aspects of this grant.<br><br>I do not think it is realistic to get ethical and HRA approval within months 1-3, particularly if you haven't recruited your staff. Who will lead on this if they are not in place? You state that you will get ethical approval from University of York. Please check this. My understanding is that patients recruitment requires NHS REC approval and not university. The team include experienced NIHR trialists so i assume this has been fully looked at.<br><br>Do you already have contact with your CAMHS you are going to recruit through? I am concerned that your staff recruitment, ethics and participant recruitment all seem to be within 4 months. I do not see this as attainable. More detail on which and how many CAMHS sites and their capacity/confidence to recruit/identify the participants is needed. Have you had discussions with your CRN to see how they may support in patient identification? Members of the research team cannot make first contact with the patients unless they are part of the patients care time.<br><br>I have noted previously that i am concerned that there needs to be flexibility in how the workshops are delivered in case of a COVID spike.<br><br>Two months for analysis also seems tight, particularly when planning to disseminate to lay and scientific audiences.<br><br>Appropriate costs and consideration have been given to IP.<br><br>As an aside, i found it confusing to have a trial co-ordinator when this isn't a trial. Perhaps a study co-ordinator?

**Plain English summary**<br>The plain English summary is intended for an interested audience, who are not necessarily specialists. The summary should be written at roughly the same level as an article in a newspaper. With this in mind, please comment on the following:<br><br>i) Does the plain English summary give a clear explanation of the research?<br>• Does it help you carry out your review? If not, why not?<br>• Is the language used appropriate and clear? If not, where are the problems?<br>• Are scientific terms, abbreviations and jargon explained? If not, which terms need explanation?<br>ii) If this research is funded, the plain English summary will be published on a variety of websites, without the rest of this application form. Could this plain English summary be used on its own to describe the proposed research? If not, what further information is needed?<br><br>Further information for researchers on how to write a plain English summary and what to include in a summary is available online at NIHR Make it clear http://www.invo.org.uk/makeitclear/.
The plain English summary was clear and reflected the overall plan. I considered the summary to avoid scientific

terms and jargon but would defer my opinion to a lay reviewer.

4. Strength of the research team

i) How well are the roles of the team members described? Is the overall team well coordinated?
ii) On the basis of track record in relevant areas, how qualified are the applicants to undertake the work using the methodologies proposed?
iii) To what extent does the research team have the necessary breadth and depth of expertise to deliver the proposed work (*e.g.* as judged by publication output and previous research funding)?
iv) How could the strength of the research team be improved?
v) If the lead applicant is inexperienced, does he/she have appropriate support (*e.g.* from their organisation and/or more senior colleagues) to deliver the work plan?

The team appears to be well co-ordinated with shared PI roles between Dr Armitt and Prof Wright. Both are experienced researchers with a track record of delivery of NIHR funded trials and a history of successful collaboration, but as the more junior member it is reassuring to see Wright mentor Armitt.

The team are further strengthened by their co-applicants ensuring that the proposed methodologies could be delivered. It is reassuring to see Dr Coventry will inform on the qualitative aspect and that there is a member of the team (Garside) who will provide PPI support. In general, the PPI and public facing aspect seems well covered by the team, with Woolley and Booth-Card demonstrating an agreed partnership with the Yorkshire Wildlife Trust, which would be key for the successful delivery of the grant. White and Hussey provide complimentary expertise on outdoor activities.

The PPI member has a lot of lived experience with ADHD, however, as a clinical co-ordinator and paramedic. I have some concerns about how well this member could be categorised as "lay". Although their knowledge, both personally and professionally will be important, a lay member can be extremely important for informing patient facing documents. The team should address how they ensure this is covered.

5. Impact of the proposed work

Dissemination, outputs and anticipated impact
NIHR aims to fund research that has the potential to be of significant benefit to the NHS, patients and the public. To support this, the applicants should consider how they will achieve impact from the outset. This helps them to identify potential impact and beneficiaries, and plan processes by which the research can directly, or incrementally over time, lead to change.

i) Have the applicants clearly expressed the problem and outlined how this research contributes towards a solution? Are the planned outputs appropriate and sufficient?
ii) Is it clear from the application who or which groups (including, as appropriate, healthcare planners, clinicians, patients and/or policy makers) are expected to benefit the most, how they will be engaged and communicated with, and if appropriate methods of engagement and dissemination are planned?
iii) Have the applicants set out appropriate activities and resources to achieve their impact goals? Is there a realistic trajectory and estimate of timescales for the benefit to reach patients/ public/ health and care services? Are there clear connections between outputs, engagement processes and impact goals?
iv) Have the applicants clearly considered what follow-on support they might need to generate or upscale impact, and how they might leverage further investment?
v) Have the applicants sufficiently and correctly identified any potential barriers they might face (e.g. IP, regulatory and acceptability) and have they properly considered how these may be overcome?
vi) Have the applicants clearly and realistically outlined the anticipated impacts, the likely scale of these impacts (both in the shorter and longer terms), and the sequence in which the impacts might occur?

The applicants have outlined how their proposed solution addresses the problem and planned appropriate methods of engagement and dissemination. I particularly like that that child friendly versions of the findings will be

co-produced by the young people. However, i am concerned that there has not been enough time allocated for this in the study plan.<br><br>I am pleased to see dissemination via their relevant partner organisations as well as academics.<br><br>The applicants mention sharing the intervention via service leads and commissioners - it will be important to engage with these partners early on and inform them of the development of the study. It is important that any developed intervention fits their needs also and this should be considered throughout the lifespan of the project.<br><br>IP has been accounted for and costed for.<br><br>Barriers have not been considered as a whole, i am unclear as to whether the applicants feel a trial should come first or implementation and real world field study. This was a bit confusing and requires further thought as to the best order of events and next steps.<br><br>I would like to see more clearly outlined the anticipated impact (it is implied that waiting lists may be shortened, but it isn't clear to me where this fits in the pathway and how this will shorten waiting lists. i.e. will receiving this mean they dont need other treatment? Or perhaps its not about shortening waiting lists but supporting on waiting lists. A clearer description of where this would fit into the pathway is needed. If this is to be explored as part of the intervention, appropriate and time and resources should be designated to this.<br><br>The timescales for the next steps and trial are appropriate.

6. Value for money

i) Taking into consideration the costs associated with undertaking the research, is there sufficient justification for the resources requested? Are all costs essential for the work proposed?<br>ii) Are appropriate resources set aside for patient and public involvement - including plans for a training and support budget?<br>iii) Where relevant, are resources to support impact - other than those for public and patient involvement - included and appropriate?<br>iv) Taking into account the total cost of the research, including the NHS costs, to what extent does the research provide value for money?<br>v) If required, are funds requested for NHS support and treatment costs appropriate and justified?
The project seems well costed. Appropriate staffing costs have been allocated as well as adequate % for co-investigators.<br><br>PPI is well costed and costs for travel and vouchers are considered.<br><br>Costs of OA fees and conference attendance are also included.<br><br>The SoECAT appears accurate and service support costs have been considered.<br><br>No further comments on costings.

7. Involvement of patients and the public

**Was there any patient and public involvement in the application?**	Yes

i) What is your assessment of the patient and public involvement (if any) in the development of the application,

including involvement in: Identifying the research topic; prioritising the research questions; preparing the application (e.g. contributing to the research design); and identifying potential impact?<br>ii) What is your assessment of any proposed plans for patient and public involvement throughout the life of the research? Can you identify particular strengths, weaknesses and/or areas for improvement?
The applicants sought advice from an appropriate PPI group (mothers of CYP with ADHD) on how to consider health and financial inequalities when planning the intervention as well as informing the recruitment strategy (although no details are provided as to what was changed as a result).<br><br>The PPI lead is a senior paramedic and thus may require further support when creating lay summaries to those with no clinical knowledge.<br><br>PPI feedback was also sought in addressing comments from Stage 1. However, it isn't clear if PPI contributed to the PPI dissemination plan or the writing of the lay summary.<br><br>Overall, PPI throughout the life of the research is well addressed with inclusion at all stages - indeed PPI is at the heart of the overall design.

8. Additional Comments

If you have any additional comments, not covered by the sections above, please provide them below.

NIHR | National Institute for Health Research

Research for Patient Benefit Review Summary

Reference Number	NIHR203043
Research Title	Development of a nature based intervention for children with ADHD (NICA)
Chief Investigator	Dr Hannah Armitt
Contracting Organisation	Leeds and York Partnership NHS Foundation Trust
Total Amount Requested	£149,599.00
Reviewer Number	4

Table Of Contents

1. Reviewer Expertise
2. Relevance of the proposed research
3. Quality of the proposed work
4. Strength of the research team
5. Impact of the proposed work
6. Value for money
7. Involvement of patients and the public
8. Additional Comments

1. Reviewer Expertise

**Please indicate the nature of your expertise by clicking on the appropriate tick box(es) below:**
Clinician in the same/a very similar field, Researcher in the same/a very similar field

If the tick boxes above do not adequately capture the nature of your expertise, please briefly provide details in the box below (or use it to give us more detail about your expertise if you wish):
I have previously supervised the dissertation of an MA student developing green interventions for veterans, so have familiarity with the concepts of nature based therapies.

2. Relevance of the proposed research

i) How relevant and important is the proposed research to the priorities and needs of the NHS, and does it offer a health/healthcare solution with demonstrable benefit to patients?<br>ii) Does the application demonstrate an awareness and understanding of previous relevant research or developments in this area?<br>iii) To what extent does the proposed research add distinct value or advance existing knowledge in this area, taking into account wider ongoing or completed research?
This application fits well with he move to social prescribing and the need to diversify treatment options for young people with ADHD.<br><br>The application demonstrates an awareness of how this fits within current knowledge of green interventions.<br><br>This application opens the way for developing novel and complementary treatments for this group of young people,

3. Quality of the proposed work

**Research Design**<br>i) How appropriate is the research design in relation to the stated objectives?<br>ii) To what extent is the proposed design and methodology for all elements of the research well defined, appropriate, valid and feasible within the timeframe and resources requested?<br>iii) What are the strengths and weaknesses of the research design as proposed?<br>iv) To what extent does the research show originality and innovation?
This is a relatively small scale design which is appropriate for the exploratory nature of the design.<br><br>The design has set itself realistic goals in terms of what in can potentially achieve within the timescale.<br><br>The design has good PPI elements, which means they need to be flexible to respond to the input of experts by experience to refine the design within the timeframe.<br><br>Nature-based interventions are novel in general, but particularly for this group of young people, and opens opportunities for novel treatment interventions.

Has the research been designed with reference to an appropriate review of the existing literature?
Yes

**Work plan and proposed management arrangements**<br>i) How appropriate are the work plan and project management arrangements, and do they give confidence that proposed milestones will be met within the specified timeframe?<br>ii) Are the necessary clinical, academic or organisational links needed to support the research, in place?<br>iii) Please assess whether the key risks identified by the applicants have been adequately addressed, such as:<br><br>• Ethical, scientific, technical and organisational challenges<br>• Intellectual Property (IP) and commercial issues<br>iv) Please identify any additional barriers to the proposed work, not mentioned in the application form, that the applicants are likely to encounter in meeting their milestones<br>v) If this application has been submitted to the NIHR Programme Grants for Applied Research Programme, what added value would be delivered over and above the dividends from the individual elements?
Work plan and organisational links seem appropriate, the PI is inexperienced at this level but has arranged support ans supervision from other staff who do have sufficient experience in these kinds of projects. Experience of managing projects to time is included in these arrangements.<br><br>Additional expertise has been put in place as necessary,<br><br>Ethical clearance for this kind of project has not yet been achieved and will be complex given the nature of the sample group. Time is allowed to ensure that this is addressed before any work starts.<br><br>Given the novel nature of this research explaining to others the specific value of nature-based interventions for treatment purposes needs to be done clearly and simply.<br><br>This study has the potential for national and international influence in adding to treatment options for young people with ADHD.

**Plain English summary**<br>The plain English summary is intended for an interested audience, who are not necessarily specialists. The summary should be written at roughly the same level as an article in a newspaper. With this in mind, please comment on the following:<br><br>i) Does the plain English summary give a clear explanation of the research?<br>• Does it help you carry out your review? If not, why not?<br>• Is the language used appropriate and clear? If not, where are the problems?<br>• Are scientific terms, abbreviations and jargon explained? If not, which terms need explanation?

ii) If this research is funded, the plain English summary will be published on a variety of websites, without the rest of this application form. Could this plain English summary be used on its own to describe the proposed research? If not, what further information is needed?<br><br>Further information for researchers on how to write a plain English summary and what to include in a summary is available online at NIHR Make it clear http://www.invo.org.uk/makeitclear/.
Plain English summary is good and clear.<br><br>It is sufficient for the additional purposes described.

4. Strength of the research team

i) How well are the roles of the team members described? Is the overall team well coordinated?<br>ii) On the basis of track record in relevant areas, how qualified are the applicants to undertake the work using the methodologies proposed?<br>iii) To what extent does the research team have the necessary breadth and depth of expertise to deliver the proposed work (*e.g.* as judged by publication output and previous research funding)?<br>iv) How could the strength of the research team be improved?<br>v) If the lead applicant is inexperienced, does he/she have appropriate support (*e.g.* from their organisation and/or more senior colleagues) to deliver the work plan?
Different roles are well described and clear, team appears well co-ordinated.<br><br>Although the PI is relatively inexperienced this is addressed with supervisory and mentoring arrangements.<br><br>Publications not included in CVs, but methodologically the team seem well qualified. There is a track record of return on previous funding for the supervisors.<br><br>No suggestions for improving the team<br><br>As noted this is a relatively inexperienced PI, but sufficient arrangements have been made for supervision and mentoring from more experienced staff.

5. Impact of the proposed work

**Dissemination, outputs and anticipated impact**<br>NIHR aims to fund research that has the potential to be of significant benefit to the NHS, patients and the public. To support this, the applicants should consider how they will achieve impact from the outset. This helps them to identify potential impact and beneficiaries, and plan processes by which the research can directly, or incrementally over time, lead to change.<br><br>i) Have the applicants clearly expressed the problem and outlined how this research contributes towards a solution? Are the planned outputs appropriate and sufficient?<br>ii) Is it clear from the application who or which groups (including, as appropriate, healthcare planners, clinicians, patients and/or policy makers) are expected to benefit the most, how they will be engaged and communicated with, and if appropriate methods of engagement and dissemination are planned?<br>iii) Have the applicants set out appropriate activities and resources to achieve their impact goals? Is there a realistic trajectory and estimate of timescales for the benefit to reach patients/ public/ health and care services? Are there clear connections between outputs, engagement processes and impact goals?<br>iv) Have the applicants clearly considered what follow-on support they might need to generate or upscale impact, and how they might leverage further investment?<br>v) Have the applicants sufficiently and correctly identified any potential barriers they might face (e.g. IP, regulatory

and acceptability) and have they properly considered how these may be overcome?<br>vi) Have the applicants clearly and realistically outlined the anticipated impacts, the likely scale of these impacts (both in the shorter and longer terms), and the sequence in which the impacts might occur?
This is an exploratory study, so has realistic goals for developing an interventions which will need further testing when it is developed. This is understood by the researchers.<br><br>Dissemination is anticipated within specialist routes for those involved in treatment of this group of young people.<br><br>Further development of this project will be needed if it proves successful, but this is difficult to anticipate at this point.<br><br>Not applicable unless this becomes a successful treatment option.<br><br>It is difficult to anticipate the potential impact until this treatment route has been evaluated fully.

6. Value for money

i) Taking into consideration the costs associated with undertaking the research, is there sufficient justification for the resources requested? Are all costs essential for the work proposed?<br>ii) Are appropriate resources set aside for patient and public involvement - including plans for a training and support budget?<br>iii) Where relevant, are resources to support impact - other than those for public and patient involvement - included and appropriate?<br>iv) Taking into account the total cost of the research, including the NHS costs, to what extent does the research provide value for money?<br>v) If required, are funds requested for NHS support and treatment costs appropriate and justified?
This project seems to offer good value for money, budget is detailled and sufficient.<br><br>PPI is embedded well in the design<br><br>Resources for PPI are detailed and sufficient.<br><br>The research represents good value for money.

7. Involvement of patients and the public

**Was there any patient and public involvement in the application?**	Yes

i) What is your assessment of the patient and public involvement (if any) in the development of the application, including involvement in: Identifying the research topic; prioritising the research questions; preparing the application (e.g. contributing to the research design); and identifying potential impact?<br>ii) What is your assessment of any proposed plans for patient and public involvement throughout the life of the research? Can you identify particular strengths, weaknesses and/or areas for improvement?
Initial PPI involvement is good, expert by experience also has knowledge of policy development within the NHS which is useful.<br><br>Further co-production involving more experts by experience is embedded in the design.

8. Additional Comments

If you have any additional comments, not covered by the sections above, please provide them below.
This is a very interesting proposal which has potential to develop additional treatment options for children and young people with ADHD.

NIHR | National Institute for Health Research

Research for Patient Benefit
Review Summary

Reference Number	NIHR203043
Research Title	Development of a nature based intervention for children with ADHD (NICA)
Chief Investigator	Dr Hannah Armitt
Contracting Organisation	Leeds and York Partnership NHS Foundation Trust
Total Amount Requested	£149,599.00
Reviewer Number	5

Table Of Contents

1. Reviewer Expertise
2. Relevance of the proposed research
3. Quality of the proposed work
4. Strength of the research team
5. Impact of the proposed work
6. Value for money
7. Involvement of patients and the public
8. Additional Comments

1. Reviewer Expertise

**Please indicate the nature of your expertise by clicking on the appropriate tickbox(es) below:**
Member of public with a more general view

If the tick boxes above do not adequately capture the nature of your expertise, please briefly provide details in the box below (or use it to give us more detail about your expertise if you wish):
BSc Hons (2:1) Forest Management.<br><br>Previously undertaken (but not completed (80 / 90 percent?) a PGCE and had many weeks of experience teaching children who suffer with ADHD.<br><br>Taught outdoor (nature based) lessons whilst a) as a trainee teacher, b) as a Ranger working for the Forestry Commission and c) as a Tree and Woodland Officer working for local Government.

2. Relevance of the proposed research

i) Is there a clear and credible reason for doing this research? If there is, what is it?<br>ii) Is this research important or relevant to patients or carers? Why is that?<br>iii) Could the results of the research make a difference to patients or carers? If yes, how would they make a difference? If not, why not?
i) There are no 'Green Treatment' options specifically catering for those with ADHD and this study has been designed to produce and refine an intervention tool to this end. The waiting lists for any 'Green Treatment' (or any ADHD intervention) are extremely long. Nature based interventions are proven methodology.<br><br>ii) From first hand experience working with SEN teachers and in the classroom with children with ADHD and speaking to their carers / parents , I would say that this kind of wholesome and holistic intervention would be welcome. Any intervention that reduces the impacts of ADHD, whilst lessening the reliance on drugs should be seriously considered.<br><br>iii) If the research produces methodology to help reduce the effects of ADHD it could be used as a tool across the board that could prove beneficial to patients and carers. The intervention may help the patient to focus, give them

more perspective, self - esteem and provide them with an interest (and outlet) not yet explored. If it could help to negate the disruptive symptoms of ADHD in some way, it would not only help the patient, but also the carers (and other children in their classroom / school)

3. Quality of the proposed work

Research Design
i) Are the outcomes the researchers are planning to measure appropriate? Will the research ultimately benefit patients, service users, carers and/or the public? Why is that the case? Are there other outcomes that are more important? If so, what are they?
ii) Have the researchers taken a realistic approach to recruiting people to participate in their research? Could this be improved and if so, how? Do you think people are likely to agree to take part? Would you be willing to take part in the research or suggest to a friend that they did?

i) The outcomes the researchers are planning to measure are appropriate in that they are planning to measure percentile of recruitment, attendance and contribution, allowing the production of an 'intervention booklet' which can then be tested in further feasibility trials. I would question though, having spent time with children with ADHD, whether this is wholly realistic, given that some children may feel that the workshops etc are just an extension of schooling (in what they may perceive as their 'free time') and the negative implications attributed to it.

I my experience I know that many children (particularly those with enhanced needs) feel that school is a chore, and would do anything to avoid any 'learning environment'. As a person with dyslexia and various learning difficulties in my childhood, I know I would have avoided any 'extra' schooling at all costs. I feel that, on this basis, the percentile of recruitment, attendance and contribution may be impacted somewhat, and that a certain amount of tolerance should be built in to this statistic as an indicator of success.

I think that the end result - the production of the methodology - the 'intervention booklet', followed by further, larger (statistically viable) feasibility trials, is the most important outcome.

ii) Given that the study is fairly small, I feel sure that the method outlined for recruitment will be satisfactory and there will be many parents / carer's and children willing to take part. As mentioned above, however, I would recommend that the 'sell' of the program to the children involved is extremely important - i.e. - the workshops must be presented in as fun a way as possible, steering away from the perception of 'lessons' and 'teaching' and focussing on phrases such as - 'getting involved with nature', 'going out for a fun day in the wild', 'Wildlife Adventure Workshop' etc. I always loved nature as a child (and hated school), I would have been excited to be involved in a program focussing on nature (with learning as a natural by product)

Has the research team taken account of previous research in this area?	Yes

Work plan and proposed management arrangements
i) How are any plans for patient and public involvement in the research also referred to in the work plan and in the proposed management arrangements? Could the plan and the management arrangements be improved from this perspective?

Plans for patient and public involvement are referred to throughout the work plan and management arrangements.

I think that maybe more detail could be given about what would happen if / when those that take part might leave the program. Would others be recruited?

Plain English summary
The plain English summary is intended for an interested audience, who are not necessarily specialists. The summary should be written at roughly the same level as an article in a newspaper. With this in mind, please comment on the following:

i) Does the plain English summary give a clear explanation of the research?

- Does it help you carry out your review? If not, why not?
- Is the language used appropriate and clear? If not, where are the problems?
- Are scientific terms, abbreviations and jargon explained? If not, which terms need explanation?

ii) If this research is funded, the plain English summary will be published on a variety of websites, without the rest of this application form. Could this plain English summary be used on its own to describe the proposed research? If not, what further information is needed?

Further information for researchers on how to write a plain English summary and what to include in a summary is available online at NIHR Make it clear http://www.invo.org.uk/makeitclear/.

i) The plain English summary did give a clear explanation of the research - it helped me by providing a brief, clear and concise overview. The language is appropriate and clear and scientific terms, abbreviations and jargon are not needed.

ii) I did feel that this could have included the actual numbers of children in each group, in order to give an overview of the scale of the study, but otherwise the Plain English summary was fine in my view.

4. Strength of the research team

i) Does the research team appear to have the right mix of skills to carry out this research? For example, if the research involves looking at what nurses do, is there a nurse on the team? If not, how could the team be strengthened?
ii) Is there one or more suitably experienced member of the research team with responsibility for coordinating, supporting and delivering patient and public involvement activities? If not, how could this be addressed?
iii) Are patients, service users or carers included in the research team? And if so, is it clear what their role or roles will be and what they will bring to the research team?

i) The mix of skills appears to be correct. The level of child psychology and 'nature based' teaching skills is appropriate, I feel.

ii) There seems to be 2 leading individuals with responsibility for coordinating, supporting and delivering patient and involvement activities - Sara Booth-Card and Kat Wooley. Natasha Green will be the single point of contact for the public contributors.

iii) It seems as though no patients, service users or carers are included in the research team, other than Natasha Green - Natasha Green is a parent of a child with ADHD. Natasha will liaise and plan PPI activities with the research team, and act as a single point of contact for all public contributors.

5. Impact of the proposed work

Dissemination, outputs and anticipated impact
NIHR aims to fund research that has the potential to be of significant benefit to the NHS, patients and the public. To support this, the applicants should consider how they will achieve impact from the outset. This helps them to identify potential benefits and beneficiaries beyond the academic community, and plan processes by which the research can directly or incrementally over time, lead to change in the ‘real world’.

i) Have the applicants clearly expressed a real-world problem and how their research contributes towards a ‘solution’? How well do the planned outputs match this aim? If not, what changes are needed?
ii) Have the applicants made it clear what impacts they are aiming to achieve from the research? Are these plans appropriate? Are they achievable? Do they seem realistic in terms of scale and timing? If not, what needs

changing?<br>iii) Have the applicants clearly stated who will benefit from this research (e.g., patients, carers, clinicians, policy makers, healthcare planners) and how they will benefit? Are plans to engage and communicate with these individuals/groups appropriate? If not, what is missing?<br>iv) Have the applicants chosen suitable activities to achieve impact? Have they made it clear how the outputs, beneficiaries and planned impacts are linked? If not, what needs changing?<br>v) Are the applicants clear on what would be needed (e.g. more funding, further partnerships) to sustain or increase impact after the project? If not, what else needs to be considered?<br>vi) Have the applicants sufficiently considered the barriers they may face in achieving impact (e.g. regulations, intellectual property/rights, acceptability to users)? Have they adequately considered how to overcome these? If not, what is missing?
i) The applicants have stated that the non medication interventions for ADHD are limited and have a long waiting list. The research is to trial interventions that utilise 'Green Treatment' ie - workshops that foster an interest and enhance understanding of the natural world, which, studies have proved, can be beneficial for all. The research is aimed at creating a protocol that can work as a suitable intervention to alleviate the symptoms of ADHD, and this information can be be disseminated to other interested parties and maybe used on a much larger scale.<br><br>ii) The clear aim of this approach is to create an intervention technique that reduces the need for medication and symptom reduction for those with ADHD. I believe these plans are appropriate and achievable. The scale and timing of the research is appropriate as a small scale trial, although I believe that, given the complexity of the issues involved, further research and refinements thereafter would be beneficial.<br><br>iii) The applicants have made it clear that carers and patients will benefit, but not explicitly stated (that I noted) the benefits to others. I feel sure that they would expect the reader to automatically understand the benefits to others (clinicians, policy makers, healthcare planners, school / teaching community) as a matter of course.<br><br>iV) The applicant is utilising various nature educational professionals who will undoubtedly use this experience to tailor - make engaging activities based on the weather / local environs etc. Having done this myself on a number of occasions I can understand how precisely defining each activity, and linking it to a planned impact is not always realistic.<br><br>v) The applicant's have not mentioned sustaining the research, or increasing scale. I believe that a larger scale project could be considered in future.<br><br>vi) This is not the kind of research that will need copywrite or worry about regulations or intellectual property - the applicants have expressed intentions on sharing the findings widely with all interested parties.

6. Value for money

The NIHR provides guidance on what can and cannot be included in the costs of research. CCF carries out an initial financial scrutiny of all applications received. A more detailed scrutiny of finances is always carried out on any application that is recommended for funding.<br>As a public reviewer, you are not expected to assess whether the entire research budget is costed correctly. However, comments on the following aspects are welcome:<br>i) Overall, does the research budget seem a reasonable investment of public money? Could it save health and social care costs in the long term?<br>ii) Are the resources set aside for patient and public involvement appropriate for the proposed activities? E.g. for training and support, travel and other expenses, staff salaries? For more see: INVOLVE's Involvement Cost Calculator. If not, how could it be improved?
i) The budget seems reasonable if a new intervention can be found to help treat the symptoms of ADHD. It will not only help with reducing costs of further treatment (and reduce the pressure on an already overloaded healthcare and intervention system), but could help with schooling for both the participant and those in his / her classroom (hopefully less disruption in the classroom). I would also assume that reduced ADHD symptoms can also improve the social engagement of the subject, improve their self esteem and mental welfare in both the short term and the future. An engagement with Nature and wildlife can only be positive and perhaps provide an interest that may lead to career options in this field in the future.

ii) The budget is relatively small compared with the benefits that might be derived from it. The expenses and staff salaries all seem reasonable, I can't find any obvious discrepancies.

7. Involvement of patients and the public

**Was there any patient and public involvement in the application?**	Yes

i) What is your assessment of the patient and public involvement in the development of the application including involvement in: identifying the research topic; prioritising the research questions; preparing the application (*e.g.* contributing to the research design); and identifying potential impact?<br>ii) What is your assessment of any proposed plans for patient and public involvement throughout the life of the research? Can you identify particular strengths, weaknesses and/or areas for improvement?
i) Apparently the development of the research question and concept for the study was discussed in initial PPI groups and 6 mothers of children aged 4-15 who had a diagnosis of ADHD attended and contributed somewhat to the research design. The PPI seems adequate, given how fluid the program of workshops is likely to be.<br><br>ii) It seems there is a PPI lead who will help coordinate activities throughout the life of the study. She is a mother of a child with ADHD and a Senior Paramedic/Clinical Coordinator with East of England Ambulance Service NHS Trust. This is definitely a strength of the proposed plans for PPI involvement. Maybe a second person could be identified for this task should she become too busy with work / illness etc.

8. Additional Comments

If you have any additional comments, not covered by the sections above, please provide them below.
I believe that this could be a very important intervention, as long as you can maintain the interest and goodwill of the children involved, and that they don't feel it's just an extension of their schooling or some kind of 'Therapy'.<br><br>I wonder if 'big' names could be brought onboard to lend it more 'kudos' in the eyes of children - someone like Bear Grylls or Steve Backshall - they only need to add a photo or two and a short statement and I know they do a lot for charities. If not with this research program, maybe as an idea for the actual interventions thereafter.<br><br>Only a small thing, but I believe if you sell the whole process to the children as a 'Wildlife Adventure' or 'Outdoor Survival Training' and other exciting phrases it would help to gain engagement.
